# TGNap1 is required for microtubule-dependent homeostasis of a subpopulation of the plant *trans*-Golgi network

**DOI:** 10.1038/s41467-018-07662-4

**Published:** 2018-12-14

**Authors:** Luciana Renna, Giovanni Stefano, Erin Slabaugh, Clarissa Wormsbaecher, Alan Sulpizio, Krzysztof Zienkiewicz, Federica Brandizzi

**Affiliations:** 1 MSU-DOE Plant Research Lab, Michigan State University, East Lansing, MI 48824 USA; 2 Department of Plant Biology, Michigan State University, East Lansing, MI 48824 USA; 3 Present Address: Department of Molecular and Structural Biochemistry, North Carolina State University, Raleigh, NC 27695 USA; 4 Present Address: Department of Molecular Genetics, The Ohio State University, Columbus, OH 43210 USA; 5 Present Address: Department of Molecular Biology and Genetics, Cornell University, Ithaca, NY 14850 USA; 6 Present Address: Department of Plant Biochemistry, Georg-August-University, Albrecht-von-Haller-Institute for Plant Sciences, 37073 Göttingen, Germany

## Abstract

Defining convergent and divergent mechanisms underlying the biogenesis and function of endomembrane organelles is fundamentally important in cell biology. In all eukaryotes, the *Trans*-Golgi Network (TGN) is the hub where the exocytic and endocytic pathways converge. To gain knowledge in the mechanisms underlying TGN biogenesis and function, we characterized TGNap1, a protein encoded by a plant gene of unknown function conserved with metazoans. We demonstrate that TGNap1 is a TGN protein required for the homeostasis of biosynthetic and endocytic traffic pathways. We also show that TGNap1 binds Rab6, YIP4 and microtubules. Finally, we establish that TGNap1 contributes to microtubule-dependent biogenesis, tracking and function of a TGN subset, likely through interaction with Rab6 and YIP4. Our results identify an important trafficking determinant at the plant TGN and reveal an unexpected reliance of post-Golgi traffic homeostasis and organelle biogenesis on microtubules in plants.

## Abstract (web summary)

The plant *trans*-Golgi network comprises functionally distinct subcompartments. Here Renna et al. show that the TGNap1 protein acts in microtubule-dependent biogenesis, tracking and function of a TGN subset, highlighting diversification within the plant TGN.

## Introduction

The *trans*-Golgi Network (TGN) is a pleiotropic network of membranes where the secretory and endocytic pathways converge. Similar to metazoan cells, the plant TGN is responsible for the sorting of cargo destined to post-Golgi compartments but also functions as an early endosome (EE)^1,2^, underscoring functional species-specific adaptations of this organelle. The TGN originates through maturation of the late Golgi cisternae^3^. While in metazoan cells the TGN is associated with the Golgi, in plant cells the TGN is largely heterogeneous with subpopulations that are either associated with the Golgi (GA-TGNs/early TGNs) or are independent from the Golgi (GI-TGNs/late TGNs). The latter are smaller and originate from the GA-TGNs^4^. Various TGN-associated proteins have been localized on overlapping, partially overlapping or separate TGNs, indicating the existence of multiple TGN subsets with distinct functions in plant cells^4^. The mechanisms underlying the functional heterogeneity of the plant TGN are yet largely unknown, but evidence for a sub-compartmentalization of the TGN membranes exist^4–8^. It has also been shown that the TGN-localized ECHIDNA (ECH) is required for secretion of the PM-associated auxin transporter AUX1, pectin, and hemicellulose, but does not influence the traffic of the PM auxin transporters PIN2, PIN3, LAX3, and ABCG11, nor endocytosis^9,10^. These results support a functional specialization of the TGN subdomains in anterograde and retrograde traffic. Adding to this complexity is the enigmatic relationship of the TGN and post-Golgi traffic with the cytoskeleton in plant cells. In metazoans, the TGN associates with a static Golgi, and the long-distance post-Golgi vesicular traffic largely depends on microtubules (MT) and MT-associated motors^11,12^. Actin is involved in post-Golgi traffic in metazoan cells but has been primarily implicated in membrane deformation for the generation of vesicles at the TGN^11,12^. In contrast, the long-range movement of plant organelles is largely dependent on actin and associated myosins^13,14^. Depletion of actin reduces post-Golgi traffic^15^, and TGNs have been found in association with actin^15,16^, further implying a functional association between TGN, post-Golgi traffic, and actin. MT and members of the kinesin MT-based motor superfamily have a role in the short-range movement of plant organelles, and mainly serve for the positioning of the MT-associated cellulose synthase compartments (MASCs) and the tracking of the cellulose synthase complex at the PM^17,18^. Thus, current knowledge infers that metazoan and plant cells have evolved distinct cytoskeleton-mediated mechanisms for post-Golgi vesicular traffic. Nonetheless, a role of MT in the recycling of the auxin transporter PIN2 from the PM through sorting nexin 1 (SNX1)-positive endosomes has been shown^19^, posing that MT may be also involved in TGN-mediated traffic through mechanisms that deserve further attention.

In this work, we report on the identification and characterization of TGNap1, a plant protein that is conserved with metazoans, interacts with MT as well as the TGN-localized Rab6 and YIP4 proteins, and is necessary for MT-dependent biogenesis and function of a TGN subset. Our findings support the existence of a marked diversification of the plant TGN membranes and establish the mechanistic foundation of an innovative model for MT-dependent requirements underlying TGN biogenesis, tracking, and function in plant cells.

## Results

### Identification of *TGNap1* in biosynthetic traffic

**Fig. 1:**
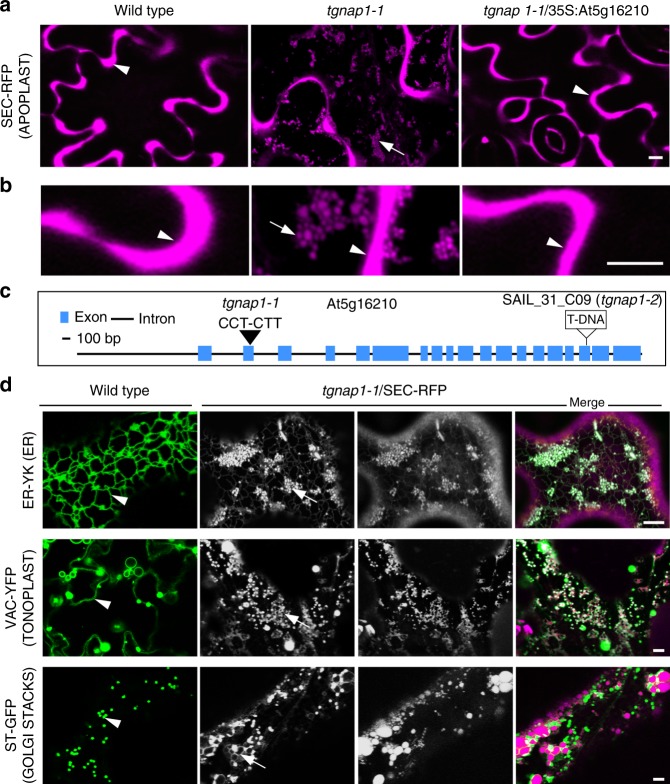
Identification, subcellular phenotypes, and mapping of *tgnap1–1*. **a** Live-cell confocal images of cotyledon epidermal cells SEC-RFP (magenta) showing distribution in the extracellular environment (apoplast, arrowheads) in WT and complemented *tgnap1–1*, but partial retention in intracellular globular structures in *tgnap1–1* (arrows). **b** Magnified views of the cellular structures presented in **a**. **c** Genomic structure of *TGNap1* (*At5g16210*). **d** Confocal images of cells of WT and *tgnap1–1*/SEC-RFP expressing markers for the ER network (arrowhead; ER-YK), vacuole membrane (arrowhead; VAC-YFP) and Golgi stacks (arrowhead; ST-GFP) (grayscale). Merged panels show in green and magenta, respectively, the endomembrane markers and SEC-RFP marker in *tgnap1–1*. In *tgnap1–1*, these markers are found also surrounding the aberrant SEC-RFP structures indicated by arrows. Scale bars = 5 µm

We conducted a confocal microscopy-based forward genetic screen on EMS mutagenized *Arabidopsis thaliana* cotyledon epidermal cells to identify mutant with partial retention of the inert bulk-flow reporter and apoplast marker, SEC-RFP^20,21^ (Fig. 1a, b). We isolated *tgnap1–1*, a mutant in which SEC-RFP was partially retained into globular structures (Fig. 1a, b) and that mapped to a C-T substitution (PRO^111^ to LEU) in *At5g16210*, a yet-uncharacterized plant locus (Fig. 1c). The gene is conserved only in multicellular organisms (Supplementary Fig. 1A) and it is homolog of the recently-identified TGN-associated KIAA1468 in mammals^22^. Linkage of *tgnap1–1* to *TGNap1* was confirmed by complementation with wild-type (WT) *TGNap1*, which reverted the phenotype of *tgnap1–1* (Fig. 1a, b). Thus, we have identified a loss-of-function mutation that partially disrupts protein secretion and maps to a plant gene of yet-unknown function.

Introgression of established markers in *tgnap1–1* allowed endomembrane characterization in the mutant. In WT cells, the ER lumen marker ER-YK was distributed to the expected ER network^23^ (Fig. 1d); however in *tgnap1–1*, the reporter was distributed in a modified ER network with a typical tubular arrangement but also enlarged globular regions overlapping with the SEC-RFP intracellular accumulations (Fig. 1d), similar to other biosynthetic traffic mutants^21,24^. Accordingly, in WT cells, the tonoplast (VAC-YFP) and Golgi (ST-YFP) markers had the expected distribution to the vacuolar and the Golgi membranes, respectively^23^; however, in *tgnap1–1* they were partially retained in a modified network of membranes enveloping the SEC-RFP globular structures (Fig. 1d). Treatment of *tgnap1–1* expressing the Golgi marker ST-GFP with brefeldin A (BFA), which redistributes Golgi membranes into the ER^25^, clearly showed that the ER membrane enveloped the intracellular SEC-RFP accumulations (Supplementary Fig. 1B). Furthermore, the YFP-RABA2a-positive TGN subpopulation showed an aberrant distribution in *tgnap1–1* compared to WT, with smaller TGNs and TGN clusters often distributed at the SEC-RFP intracellular structures (Supplementary Fig. 1C). Therefore, homeostasis of the biosynthetic pathway is compromised in *tgnap1–1*. Allelic linkage of a loss of function *At5g16210* locus and *tgnap1–1* was confirmed through crosses of an *At5g16210* transcriptional null allele (*tgnap1–2*; Fig. 1c and Supplementary Fig. 2A), which showed maintenance of the SEC-RFP mis-distribution in the *tgnap1–1* x *tgnap1–2* F1 progeny, as well as aberrant SEC-RFP distribution in transgenic *tgnap1–2* (Supplementary Fig. 2B). We used *tgnap1–2* for subsequent analyses to avoid potential offsite EMS mutations. We then conducted analyses of mucilage secretion and carbohydrate content of the alcohol insoluble residues (AIR) of the cell wall, which revealed a reduced mucilage secretion from the *tgnap1–2* seed coat (Supplementary Fig. 2C), and a reduced content of rhamnose and arabinose in the cell wall (Supplementary Fig. 2D), indicating a disrupted secretion of extracellular polysaccharides in the mutant. These results support that the general secretory traffic is partially disrupted by the loss-of-function mutations of *TGNap1* and imply that TGNap1 is required for integrity of anterograde traffic through the biosynthetic pathway.

### TGNap1 is partially required for endocytosis

**Fig. 2:**
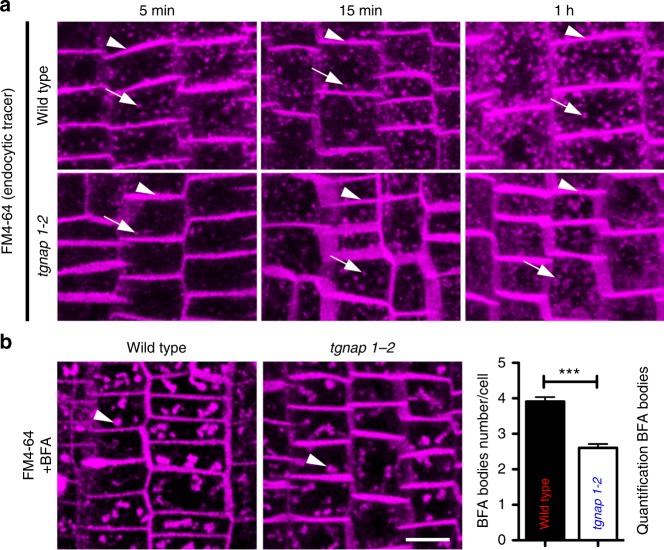
TGNap1 is required for endocytosis homeostasis. **a** Max intensity projections of serial confocal images (depth: 35 µm) of WT and *tgnap1–2* primary root cells at 5 min, 15 min, and 1 h after labeling with FM4–64. Arrowheads: FM4–64 at the PM; arrows: FM4–64 at the endosomes. **b** Confocal images of BFA bodies (arrowheads) in WT and *tgnap1–2* cells labeled with FM4–64 for 5 min. Graph indicates the number of BFA bodies/cell. WT cells (*n* = 105), *tgnap1–2* cells (*n* = 125). Scale bars = 5 µm. Error bars indicate S.E.M; Student *t*-test was applied, *p*-values are represented by asterisks when significantly different from the corresponding control: **0.001 > *p* < 0.01; ***p < 0.001. Source data are provided as a Source Data file

To test endocytosis integrity in *tgnap1–2*, we followed FM4–64 internalization and quantified BFA bodies formation^26,27^. FM4–64 internalization resulted slower in *tgnap1–2* compared to WT, as supported by a lower number of labeled endosome over time (Fig. 2a). Consistent with this, *tgnap1–2* showed a statistically lower number of BFA bodies compared to WT (Fig. 2b). Similarly, a GFP fusion to the TGN SNARE Syp61^28,29^ (GFP-Syp61) was localized to a smaller number of BFA bodies in *tgnap1–2* mutant compared to WT (Supplementary Fig. 3A, B). Furthermore, analyses of the distribution of auxin, whose distribution depends on the homeostasis of endocytosis of auxin carriers^30,31^, using the reporter DR5-GFP, whose levels correlate with auxin abundance^32,33^, revealed higher fluorescence intensity in *tgnap1–2* root tips compared to WT (Supplementary Fig. 4A). Over-accumulation of auxin reduces primary root growth^34^, in agreement with a reduced growth of the primary root of the *tgnap1* alleles compared to WT (Supplementary Fig. 2E), and a verified phenotype for lateral root density, another hallmark of imbalance in homeostasis in auxin distribution in the root^35^, in *tgnap1–2* (Supplementary Fig. 4B). The lateral root density phenotype was restored in *tgnap1–2* by supplementation with indole-3-acetic acid (IAA), an auxin analog^36^ (Supplementary Fig. 4B). The primary root growth of *tgnap1–2* was more sensitive to the auxin efflux inhibitor N-1-naphthylphthalamic acid (NPA)^37^, which hampers vesicular trafficking and endocytosis^38^ (Supplementary Fig. 4C). Furthermore, the auxin efflux carrier PIN2, which is normally distributed at polar basal and anticlinal membrane in epidermal, cortical as well as lateral cap cells of the root, and at the outer anticlinal side of root cortical cells, and is mislocalized and secreted by defective endocytosis^39^, showed statistically lower levels at the *tgnap1–2* PM compared to WT (Supplementary Fig. 4D). Also, we found fewer PIN2-GFP BFA bodies in *tgnap1–2* compared to WT (Supplementary Fig. 4E), further supporting that endocytosis is partially hampered in the *tgnap1* mutant. However, treatment with cycloheximide (CHX) led to a reduction of BFA body number in WT and more conspicuously in the mutant (Supplementary Fig. 4F). Measurements at the vacuole of dark-treated seedlings^19^ indicated reduced PIN2-GFP levels in the mutant compared to WT (Supplementary Fig. 4F). Therefore, TGNap1 availability is required for the homeostasis of the exocytic and endocytic pathways.

### TGNap1 localizes and functions at the TGN

**Fig. 3:**
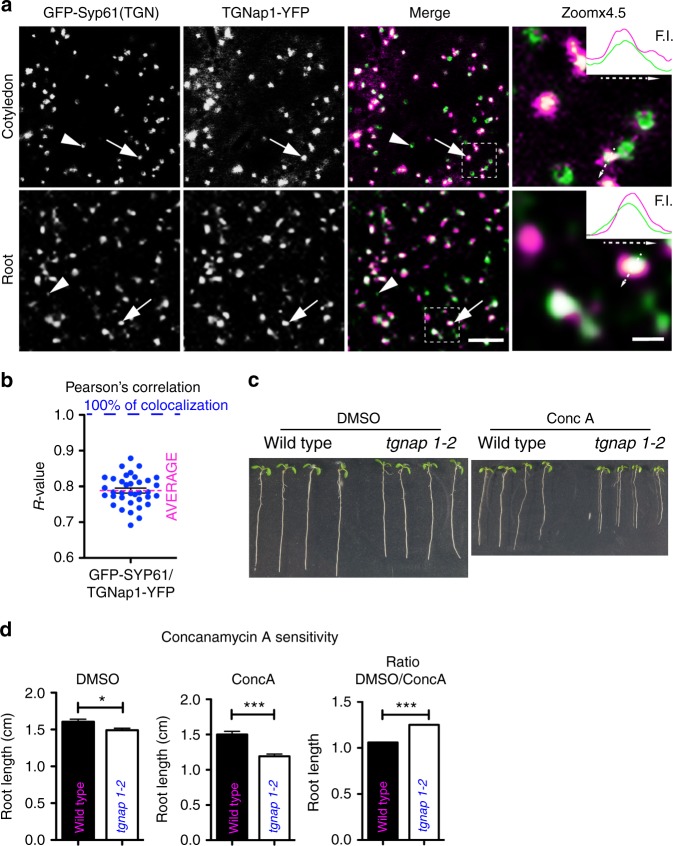
TGNap1 is a functional TGN protein. **a** Confocal images of epidermal cells coexpressing GFP-Syp61 and TGNap1-YFP (respectively, green and magenta, in white overlapping regions, single panels in grayscale). Arrows: TGNs positive for both GFP-Syp61 and TGNap1-YFP; arrowheads: Syp61-positive TGNs devoid of TGNap1-YFP signal. Scale bar = 5 µm. Zoomx4.5 panels: 4.5x magnification of the regions marked in merge (white dashed lines). Scale bar = 1 µm. Insets: intensity profile (F.I.) measurements for GFP-Syp61 (green) and TGNap1-YFP (magenta) along the arrow on individual TGNs, indicating non-complete overlap of the two signals. **b** Pearson’s correlation coefficient (*R*-value: 78.85%; *n* = 36). **c** Images of WT and *tgnap1–2* seedlings grown on DMSO or 50 nM ConcA-containing media. **d** Primary root length and relative ratio. Measurements from three independent experiments. Error bars indicate S.E.M; Student *t*-test was applied, *p*-values are represented by asterisks when significantly different from the corresponding control: *0.01 > *p* < 0.05; ***p < 0.001. Source data provided as a Source Data file

A TGNap1-YFP fusion (TGNap1-YFP), which complemented the phenotypes of root elongation (Supplementary Fig. 2E) and lateral root density (Supplementary Fig. 4B) of *tgnap1–2*, was distributed at punctate structures distinct from Golgi stacks (Supplementary Fig. 5A). Coexpression analyses with the TGN-localized Syp61 or HLB1^5^, which partially colocalize at the TGN, indicated that TGNap1 is distributed to the TGN (Fig. 3a, b and Supplementary Fig. 4B)^5^. Noticeably, TGNap1-YFP was distributed to GFP-Syp61-containing TGNs although Syp61 TGNs devoid of TGNap1 were also found (Fig. 3a). The overlap of TGNap1 and HLB1 at the TGN was also partial, with TGNs labeled exclusively by either protein fusion (Supplementary Fig. 5B). These observations were supported by Pearson’s correlation coefficients analyses^40^ (Fig. 3b and Supplementary Fig. 5C), which indicated an average of signal overlap of about 80% and 50% for the TGNap1/Syp61 and TGNap1/HLB1 combinations, respectively. Therefore, TGNap1 is distributed to a subset of TGNs. Furthermore, pixel intensity scan analyses of the fluorochrome signals across the TGN structures showed partial overlap of the TGNap1-YFP signal with either Syp61 or HLB1 fluorescent fusions (Fig. 3a and Supplementary Figs. 5D and  6A, B), indicating a partial distribution of TGNap1 to TGN subdomains devoid of Syp61 and HLB1.

To test a functional role of TGNap1 at the TGN, we assayed the sensitivity of *tgnap1–2* to concanamycin A (ConcA), a specific inhibitor of vacuolar-type H + −ATPase activity known to disrupt TGN function and inhibition of primary root growth^1,41^. Although compared to WT, *tgnap1–2* had shorter primary root elongation on DMSO (dimethyl sulfoxide; ConcA solvent), it resulted hypersensitive to ConcA (Fig. 3c, d). These results indicate that TGNap1 is a TGN protein necessary for the proper function of at least a subpopulation of TGNs.

### TGNap1 interacts with RAB6 and YIP4 at the TGN

**Fig. 4:**
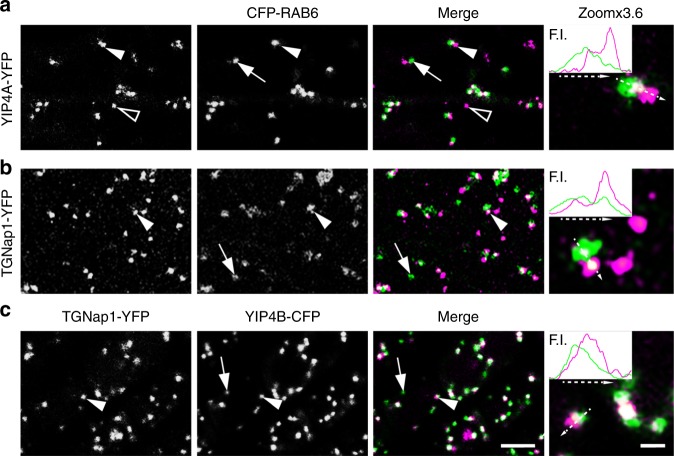
TGNap1 partially colocalizes with Rab6 and YIP4 at the TGN. **a** Confocal images of cotyledon epidermal cells expressing YIP4A-YFP and CFP-Rab6 showing co-distribution to the same organelles (arrowheads) or distinct organelles (arrow: Rab6-only positive organelle, likely Golgi stack (see Supplementary Fig. 7); empty arrow: YIP4A-only positive organelle). Insets: line intensity profile (F.I.) along the arrow on the TGN structure. Scale bars = 5 µm (main images), 1 µm (zoomed panels.) **b**, **c** Confocal images of cotyledon epidermal cells expressing TGNap1-YFP and either CFP-Rab6 (**b**) or YIP4B-CFP (**c**) showing a shifted distribution of TGNap1 vs. either Rab6 or YIP4 (arrowheads). Magnification is presented in Zoomx3.6 panels. Insets: line fluorescent intensity profile (F.I.) along the arrows with dashed lines drawn on TGN. Arrows: Rab6 (**b**) or YIP4 not colocalizing structures with TGNap1-YFP (**c**)

**Fig. 5:**
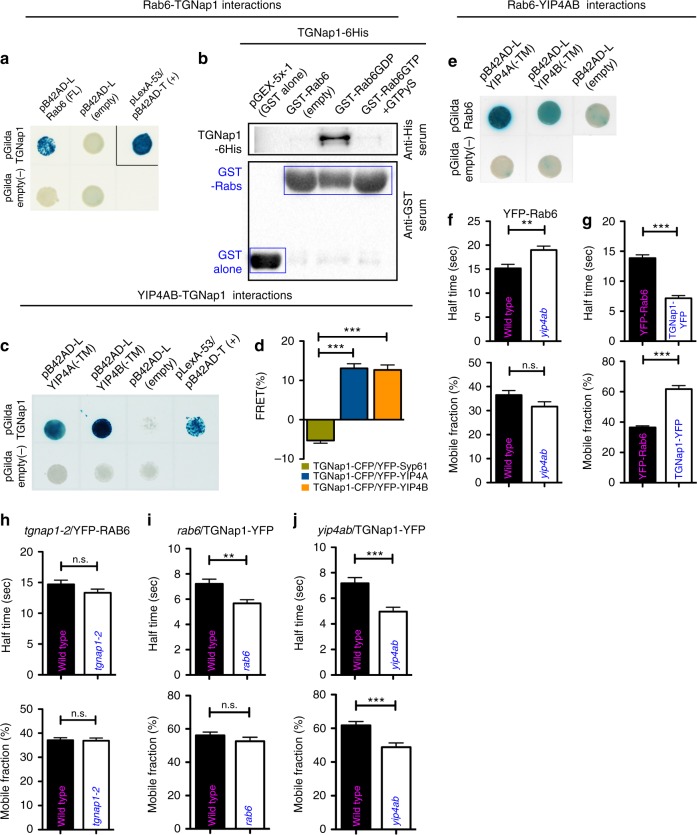
TGNAp1, RAB6, and YIP4A and B interactions and dynamics. **a** Y2H assay between TGNap1 and Rab6 (blue colonies: positive interaction, white colonies: negative controls). **b** Western blot on pull-down analyses between TGNap1 and Rab6 (anti-GST lanes: loading control). **c** Y2H assay between TGNap1 with YIP4A and YIP4B. **d** FRET analyses between TGNap1-CFP and either YFP-Syp61 (negative control) (*n* = 47) or YFP-YIP4A (*n* = 39), and YFP-YIP4B (*n* = 46) at the TGNs. **e** Y2H assay between Rab6 with YIP4A and YIP4B. **f** FRAP analyses on YFP-Rab6 in WT (*n* = 46) and *yip4ab* (*n* = 51). **g** FRAP analyses on TGNap1-YFP (*n* = 58; half-time = 7.18 ± 0.43) and YFP-Rab6 (*n* = 59; half-time = 15.18 ± 0.85). *n* = number of independent data points. **h** FRAP analyses on YFP-Rab6 in WT and *tgnap1–2* showing no significant differences in the dynamics in the two backgrounds, respectively: *n* = 55; half-time = 14.69 ± 0.70 and *n* = 55; half-time = 13.32 ± 0.60. **i** FRAP analyses on TGNap1-YFP in WT and *rab6*. In WT, TGNap1-YFP cycles on and off the TGN membranes with a half-time = 7.21 ± 0.38 (*n* = 49); however, when the cellular abundance of RAB6 is reduced compared to WT, TGNap1-YFP cycles at a significantly higher rate (*n* =52; half-time = 5.67± 0.29). **j** FRAP analyses on TGNap1-YFP in WT and *yip4ab* show a significant increase in the cycling of TGNap1-YFP on and off the TGN membranes when YIP4 is not available (*n* = 47; half-time = 4.96 ± 0.34) compared to WT (*n* = 59; half-time = 7.18 ± 0.43). Error bars indicate S.E.M; Student *t*-test was applied, *p*-values are represented by asterisks when significantly different from the corresponding control: ***p < 0.001; **0.001 > *p* < 0.01; n.s., not significant. Source data are provided as a Source Data file

To gain insights on TGNap1 function at a molecular level, we aimed to identify putative interactors for TGNap1. We conducted a yeast two-hybrid (Y2H) screen with TGNap1 as bait against an *Arabidopsis* cDNA library. Among the candidate interactors (total number of blue colonies/hits: 36), we found YIP4A (4 hits), YIP4B (3 hits), and Rab6A/RABH1B (herein indicated as Rab6; 2 hits). YIP4A and YIP4B are multispanning membrane proteins of yet-unknown function with high amino-acid sequence identity (85%) that colocalize and operate redundantly at the TGN^1,41^. We established that, in addition to the Golgi^42^ (Supplementary Fig. 7), CFP-Rab6 was distributed to the TGN, as supported by colocalization analyses with YIP4A (Fig. 4a and Supplementary Fig. 6C). We also found that TGNap1-YFP was distributed to Rab6- and YIP4-positive TGNs, although Rab6 was distributed to other structures devoid of TGNap1 (Fig. 4b, c), which likely comprise Golgi stacks. The distribution of TGNap1 partially overlapped with that of Rab6 and YIP4 at the TGN (Fig. 4b, c and Supplementary Fig. 6C), supporting our initial findings that TGNap1 is localized at TGN subdomains. A partial co-distribution of TGNap1 with Rab6 and YIP4 at the TGN is consistent with our initial Y2H interaction results, which were confirmed by a direct Y2H assay with Rab6 (Fig. 5a) and an in vitro pull-down with recombinant proteins (Fig. 5b), which established that TGNap1 binds preferentially inactive Rab6 (Fig. 5b). Through a direct Y2H analysis of TGNap1 with the N-terminal cytosolic domain of either YIP4A or YIP4B, we confirmed interactions for the TGNap1-YIP4 pairs (Fig. 5c). The interactions were further confirmed in vivo through fluorescence resonance energy transfer (FRET) with YIP4A and YIP4B (Fig. 5d). We also established an interaction of Rab6 and the N-terminal cytosolic domain of each YIP4 isoform (Fig. 5e).

Next, to uncover functional insights in the protein–protein interactions established thus far, we performed fluorescence recovery after photobleaching (FRAP) analyses to measure the rate of exchange of proteins on and off membranes. In WT, Rab6 cycled on and off membranes with a 15.18 ± 0.85 s half-time, but in *yip4ab* mutant^41^ it cycled significantly more slowly (half-time: 19.01 ± 0.78 s; 25% reduction) (Fig. 5f). Furthermore, we established that TGNap1-YFP cycled on and off the TGN membranes faster than Rab6 (half-time: 7.18 ± 0.43 s) (Fig. 5g) and that the loss of *TGNap1* did not cause noticeable changes in the subcellular distribution of YFP-Rab6 and the cycling of YFP-Rab6 on and off the TGN membranes (Fig. 5h and Supplementary Fig. 8A). However, a partial loss of Rab6 (Supplementary Fig. 7B) caused an increased turnover (i.e., 27% faster) of TGNap1-YFP on and off the TGN membranes (half-time = 5.67 ± 0.29), compared to WT (half-time = 7.21 ± 0.38) (Fig. 5i). Next, to understand the dependence of the TGNap1 binding at the TGN on YIP4, we performed a FRAP experiments in *yip4ab* lines expressing TGNap1-YFP. We found that the loss of YIP4 influenced the permanence time of TGNap1 at the membrane (half-time = 4.96 ± 0.34), which was significantly reduced (i.e., 44%) compared to WT (half-time = 7.18 ± 0.43) (Fig. 5j). Therefore, YIP4, Rab6, and TGNap1 functionally interact at the TGN. Although TGNap1 is not required for the localization and dynamics of Rab6 to the TGN membranes, Rab6 and YIP4 are required for the homeostatic permanency of TGNap1 on the TGN membrane.

### TGNap1 is a MT-binding protein

**Fig. 6:**
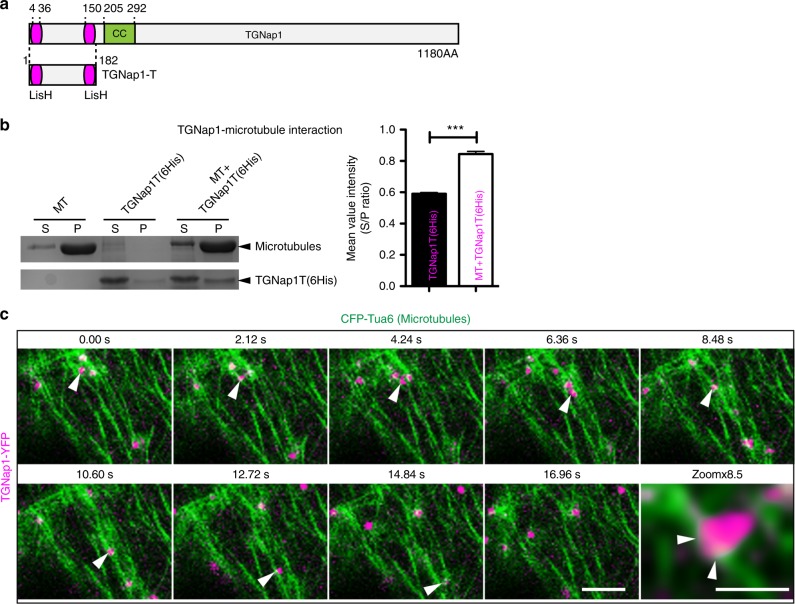
TGNap1 is a MT-binding protein. **a** TGNap1 protein domains showing LisH and coiled coil (CC) domains. Numbers indicate domain positions and total protein length (AA: amino acid residues). TGNap1-T indicates the LisH domain-containing region used for the interaction assay presented in **b**. **b** MT sedimentation assay with recombinant TGNap1T-His and bovine brain MT showing that TGNap1 is in supernatant in the absence of MT (S), and precipitates in the pellet (P) in the presence of MT. The graph represents the soluble/ pellet ratio of the mean three measurements value for TGNap1 protein band intensity. **c** Images extracted from confocal microscopy time-lapse (see Supplementary Movie 1) acquired on cotyledon epidermal cells coexpressing TGNap1-YFP (magenta) and CFP-Tua6 (green). Arrowheads point to one TGN that was tracked overtime showing a continuous association with MT. Zoomed image shows a close-up of the same TGN; arrowheads indicate points of contact of the TGN with MT. Scale bars = 5 µm (main images); 1 µm in zoomed image. Student *t*-test was applied, *p*-values are represented by asterisks when significantly different from the corresponding control: ***p < 0.001; **0.001 > *p* < 0.01; n.s., not significant. Source data is provided as a Source Data file

Next, we carried out TGNap1-YFP pull-downs with anti-GFP serum followed by LC/MS-MS analyses for identification of additional binding partners. These comprised three vacuolar ATPase subunits, including the TGN-localized vacuolar ATP synthase subunit A^1^ (Supplementary Fig. 9), consistent with TGN association of TGNap1, and the PM and TGN-localized clathrin heavy chain (CHC)^43,44^ (Supplementary Fig. 9). A YFP-CHC fusion^44^ colocalized with TGNap1-CFP (Supplementary Fig. 10A). We also identified tubulin 3 and 4 (Supplementary Fig. 9). Scanning analyses of the TGNap1 amino-acid sequence with ELM (Eukaryotic Linear motif)^45^ predicted two LisH domains in the N-terminus of TGNap1 (Fig. 6a). LisH domains are found in MT-interacting proteins and facilitate direct protein interaction with MT^46^. We performed an in vitro MT-binding assay with recombinant His-TGNap1-LisH domains (TGNap1T; amino acid: 1–182; Fig. 6a). We found that TGNap1T was predominantly in the soluble fraction when alone in the reaction; however, in the presence of MT, the levels of TGNap1T precipitated in the pellet increased significantly (Fig. 6b). Therefore, TGNap1 associates with MT in vitro directly, although the results do not exclude the possibility that additional factors absent from the in vitro reaction may modulate the interaction in vivo. We overexpressed TGNap1-YFP in the presence of CFP-TUA6, a functional tubulin marker^47,48^, in tobacco and found a localization to punctate structures, likely the TGNs, cytosol and along MT (Supplementary Fig. 11A), posing that TGNap1 binds MT not only in vitro but also in vivo. We then assayed the sensitivity of *tgnap1–2* to chemical MT-depolymerization by oryzalin^49^, which leads to radial swelling of the root^50^. We found a significant increase of the *tgnap1–2* root tip diameter compared to WT (Supplementary Fig. 10B), in support of a functional relationship between TGNap1 and intact MT. Overexpression of TGNap1 did not affect plant growth and resistance to oryzalin compared to WT (Supplementary Figs. 2F,G and 10B), supporting the hypothesis that the function of TGNap1 relies on mechanisms that are saturable.

Dividing root cells showed a marked accumulation of TGNap1-YFP-TGNs at the cell plate marked by KNOLLE^51^ (Supplementary Fig. 11B). The notion that MT drive cell plate formation^52^ and the physical and functional association of TGNap1 with MT demonstrated thus far prompted us to test an association of TGNap1-TGNs with MT in time-lapse microscopy analyses. The TGNap1-positive TGNs exhibited prolonged and close association with CFP-Tua6-labeled MT (Fig. 6c and Supplementary Movie 1), supporting the hypothesis that TGNap1 could function as a linker between the TGN and MT allowing the tracking of TGNs on the MT. We then treated TGNap1-YFP lines with oryzalin and followed the TGN movement via live-cell imaging. Oryzalin caused clustering and reduced movement of the TGNs (Supplementary Fig. 11C). These subcellular phenotypes occurred to a much lesser extent in cells treated with the actin depolymerizing drug latrunculin B^53^ (Supplementary Fig. 11C, D). The oryzalin treatment also caused a partial redistribution of TGNap1 to the cytosol and to the appearance of numerous small punctate structures (Supplementary Figs. 10D and 11D), supporting that MTs integrity is necessary to maintain the subcellular distribution of TGNap1 and TGNap1-associated membranes.

**Fig. 7:**
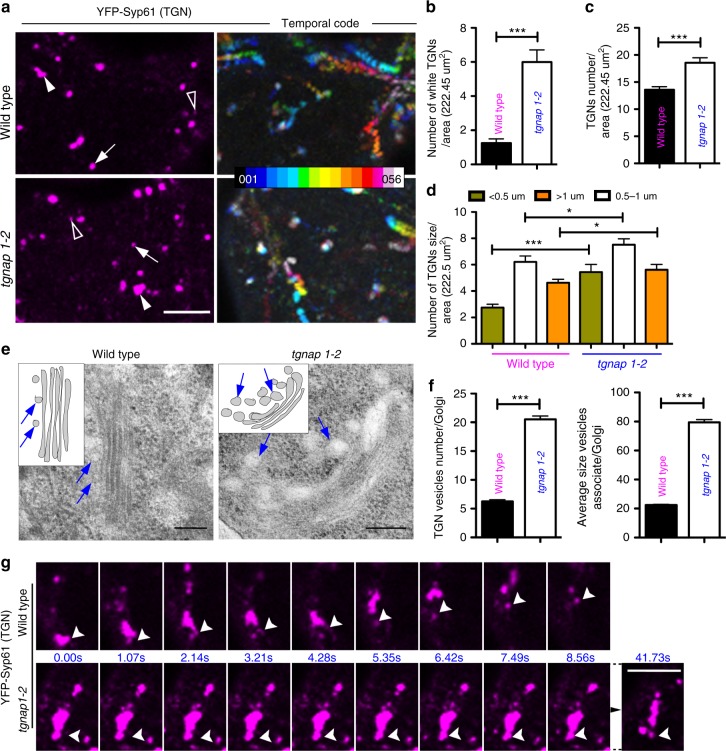
Streaming and biogenesis of a TGNs subset requires TGNap1. **a** Confocal images of YFP-Syp61-positive TGNs in cotyledon epidermal cells of either WT or *tgnap1–2*. Images are either single (left panels in magenta) or compound (right panels, color gradient images). In single images, arrowheads, arrows, and empty arrowheads indicate large, medium, and small TGNs, respectively. Compound panels are composite of 56 frames each pseudocolored along a color gradient (see scale) captured at 1.05 s interval. White color (last frame) overlapping other colors denotes slow mobility of the TGNs. Scale bar = 5 µm. **b** Quantification of the number of YFP-Syp61 TGNs pseudocolored white in cotyledon epidermal cells of either WT or *tgnap1–2* background (Student *t*-test was applied). **c** Quantification of the number of total YFP-Syp61 TGNs in cotyledon epidermal cells in WT and *tgnap1–2* background, expressed as number of white TGNs/222.5 µm^2^ area (*n* = 50 areas/genotype). **d** Size distribution of YFP-Syp61 TGNs in cotyledon epidermal cells of either WT or *tgnap1–2* (*n* = 50 areas/genotype) (*p*-value calculated with one-way Anova with Tukey’s post test). **e** Transmission electron micrographs of Golgi and TGNs (arrows) in WT and *tgnap1–2* roots. Insets: diagrams representing TGN-GA structures identified in the images. Scale bars = 0.2 µm. **f** Quantification of number and size of GA-TGN vesicles/Golgi (*n* = 60). Error bars: S.E.M. *** = *p* < 0.001 (Student *t*-test was applied, *p*-values are represented by asterisks when significantly different from the corresponding control). Number of Golgi analyzed = 60. **g** Frames from time-lapse microscopy (see Supplementary Movie 2A, B) of YFP-Syp61 labeled TGNs in cotyledon epidermal cells of WT and *tgnap1–2* background showing GI-TGNs biogenesis (arrowheads) over time. Numbers between panels express the time (seconds). Note the delay of GI-TGN detachment in *tgnap1–2* compared to WT. Scale bar = 1 µm. Source data is provided as a Source Data file

We next tested if the availability of TGNap1 on the TGN membranes could influence a MT-mediated movement of TGNs and analyzed the movement of TGNs in WT and *tgnap1–2* cells expressing YFP-Syp61. The conspicuous movement of TGNs usually found in WT cells was attenuated in *tgnap1–2* (Supplementary Movie 2A, B). This was confirmed by frame-based temporal color code analyses (i.e., black-to-white pseudo-color gradient)^54^, which showed that the TGNs in WT cells were distributed along long strings (Fig. 7a, b) indicating highly motile TGNs. In contrast, the TGNs in *tgnap1–2* were more abundantly distributed along shorter colored strings predominantly pseudocolored white (Fig. 7a, b). Therefore, the loss of TGNap1 negatively affects TGN motility, in accordance with our initial hypothesis for a role of TGNap1 in TGN movement. However, the presence of motile TGNs of *tgnap1–2* cells indicates that this effect is linked only to a subpopulation of TGNs. To test an involvement of Rab6 and YIP4 in MT-associated processes we assayed the sensitivity of *rab6* and *yip4ab* to oryzalin, and found hypersensitivity compared to untreated roots (Supplementary Fig. 10C). Nonetheless, Rab6 does not interact with MT (Supplementary Fig. 10E). These results imply a role of Rab6 and YIP4, which interact with TGNap1, in MT-guided processes that are independent from a direct Rab6-MT interaction.

### TGNap1 controls TGN biogenesis in a MT-dependent fashion

As GI-TGNs generate from GA-TGN^4^, we next explored a role of TGNap1 on TGN maturation and biogenesis by analyzing the number and size of YFP-Syp61 TGNs in WT and *tgnap1–2*. The TGNs labeled by YFP-Syp61 are heterogeneous in size, with larger TGNs corresponding to the GA-TGNs and smaller TGNs being the GI-TGNs^4^ (Fig. 7a). We discovered that the number of TGNs was higher in *tgnap1–2* compared to WT (Fig. 7c). Next, we assayed for TGN size differences between WT and *tgnap1–2*. We arbitrarily set three size groups based on TGN diameter: large ( > 1 µm), medium (0.5–1 µm) and small TGNs ( < 0.5 µm), and counted the number of TGNs fitting in each category. We found a significant increase of small, medium, and large size TGNs in *tgnap1–2* compared to WT (Fig. 7d), and noted that the larger TGNs are reminiscent of clusters of membranes (Fig. 7a, g). Therefore, TGNap1 is required to maintain the morphology and number of TGNs in the cell; its loss affects the biogenesis of TGNs from existing TGNs and possibly the delivery/fusion of TGN vesicles with target membranes. We next carried out ultrastructure analyses of the TGN-Golgi complex. In WT, we found the expected stacked structure of the Golgi cisternae with the GA-TGN juxtaposed to the *trans*-cisternae (Fig. 7e). However, in *tgnap1–2*, we found large blebbing profiles at the late Golgi cisternae and at the GA-TGN (Fig. 7e), which reminisced the appearance of disrupted TGNs in cells treated with ConcA^55^. Quantitative analyses showed a significant increase in number and size of Golgi associated-vesicles in *tgnap1–2* compared to WT (Fig. 7e, f). We conclude that TGNap1 availability is required to maintain the structural integrity of the GA-TGN and, possibly, the biogenesis of TGNs from other TGNs. The latter possibility was confirmed through high-resolution confocal time-lapse microscopy in WT and *tgnap1–2* cells expressing YFP-Syp61. We found that in WT cells TGNs could bud from the larger TGNs rapidly; in marked contrast, this process was considerably delayed in *tgnap1–2* (Fig. 7g). Because in the absence of TGNap1 the size and the overall number of TGNs increases compared to WT, these results support the hypothesis that TGNap1 is required for the biogenesis of a subset of TGNs from existing TGNs but also for their depletion over time (e.g., fusion with target membranes).

**Fig. 8:**
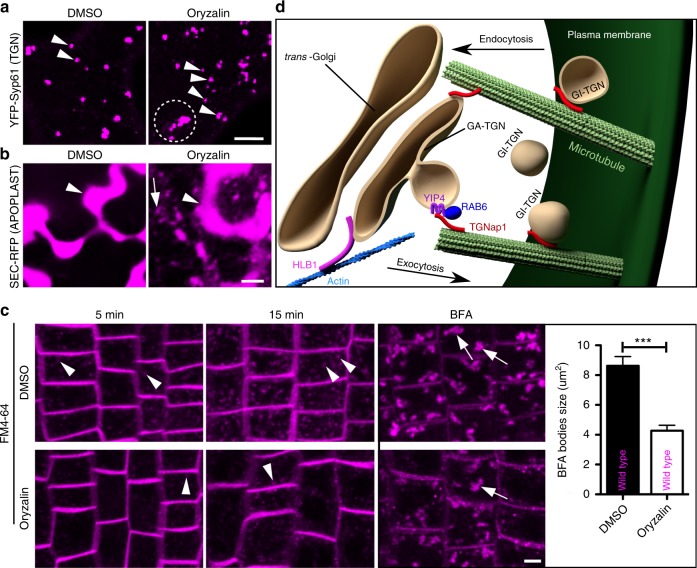
The loss of MT phenocopies the *tgnap1* subcellular phenotypes. **a** Confocal images of TGNs labeled by YFP-Syp61 in WT cotyledon epidermal cells untreated (DMSO) or treated with oryzalin 10 µm for 7 h showing appearance of small TGNs (arrowheads) and clustered TGNs (circled with dotted line) in the treated sample compared to the untreated one. **b** Effect of oryzalin treatment on cotyledon epidermal cells expressing SEC-RFP. The marker is in the apoplast (arrowheads) in untreated and treated cells, but also accumulates in intracellular structures (arrow) in treated cells. **c** Internalization of FM4–64 in WT root cells untreated (DMSO) or treated with oryzalin at 5 min and 15 min. Arrowheads indicate endosomes. Arrows in confocal images indicate BFA bodies, graph indicates BFA bodies size in oryzalin untreated and treated WT plants. WT untreated (DMSO) BFA bodies (*n* = 100), WT treated (oryzalin) BFA bodies (*n* = 80). Scale bars = 5 µm. Error bars indicate S.E.M. Student *t*-test was applied, *p*-values are represented by asterisks when significantly different from the corresponding control *** = *p* < 0.001. **d** Model for a role of TGNap1 at the TGN. TGNap1 associates to subdomains of a subpopulation of TGNs in plant cells, where it influences biogenesis and function of the TGN as well as the movement of GI-TGN/secretory vesicles through MT. The role of TGNap1 in exocytosis involves Rab6 and YIP4, either as a ternary complex or through sequential protein–protein interactions, but its role in endocytosis is likely independent from YIP4, given the lack of a requirement of YIP4 in endocytosis^41^. Schematic models were made and rendered in the 3D rendering application, Blender (www.blender.org), and further modified (addition of text and formatted for publication) using the vector-graphics editor Inkscape (inkscape.org)

Given the functional relationship of TGNap1 with MT, we hypothesized that MT could be involved in the verified defects of the TGN in *tgnap1*. Therefore, we next tested whether a chemical depletion of MT could at least partially phenocopy the loss of TGNap1 at a cellular level. We treated cells expressing YFP-Syp61 with oryzalin and found increased appearance of TGN clusters and small TGNs compared to untreated cells (Fig. 8a). This phenotype recalled the appearance of YFP-Syp61 TGNs in *tgnap1–2* (Fig. 7a). We then tested the distribution of SEC-RFP upon treatment with oryzalin and found that, similar to the *tgnap1* alleles, SEC-RFP was partially retained inside intracellular globular structures (Fig. 8b), highlighting at least a partial dependence of exocytosis on MT. Finally, we monitored the internalization of FM4–64 in cells of untreated and oryzalin-treated roots. We found that in treated cells, the internalization of the dye was delayed, as supported by a reduced labeling of endosomes compared to untreated control (Fig. 8c), indicating that the loss of MT causes defects in endocytic internalization, as further confirmed by a reduced appearance of BFA bodies in treated cells compared to untreated control (Fig. 8c). Altogether these results indicate that the loss of MT affects the morphology and function of the TGNs at the intersection of the exocytic and endocytic pathways. As the loss of MT partially phenocopies *tgnap1–1* subcellular phenotypes and TGNap1 binds MT, we conclude that the defects in TGN morphology and function established in *tgnap1* depend on a functional interaction of TGNap1 with the MT cytoskeleton.

## Discussion

The identification and characterization of proteins and protein complexes at the plant TGN is one of the main challenges in understanding the dynamic nature and the functional complexity of this organelle. In this work, we report on the identification and characterization of TGNap1, a conserved element of the plant TGN proteome that is required for the biogenesis and function of this organelle in the biosynthetic and endocytic pathways. Our results indicate a MT-binding ability of TGNap1 and a MT requirement for the proper homeostasis of TGN-mediated traffic. Altogether our findings support a MT-based model for the biogenesis and function of a subset of TGNs in plants, which at least partially hinges upon the function of TGNap1 as a linker between the TGN membrane and the MT cytoskeleton (Fig. 8d).

Loss of ECHIDNA, a YIP4-interactor of unknown function, affects secretion but not endocytosis^41^. TGNap1 is required for the homeostasis of secretion and endocytosis, indicating a functional specialization of the plant TGN proteome in the endomembrane traffic. TGNap1 may facilitate the targeting of endocytic vesicles to the TGN or influence endocytosis indirectly by contributing to anterograde traffic from the TGN.

A *tgnap1* null is viable, supporting a non-essential role of TGNap1 in traffic by sharing overlapping roles with other proteins on the same membranes or by assuming independent roles in general traffic homeostasis. The evidence that, similarly to TGNap1, the loss of the putative actin-binding TGN protein HLB1 partially compromises exocytosis and endocytosis^5^ supports the latter model. However, HLB1- and TGNap1-containing TGNs do not completely overlap. Therefore, in plant cells multiple exocytic and endocytic pathways may co-exist and may be controlled by functionally distinct proteins populating TGN subsets. A diversification of traffic pathways and their regulators may be advantageous to ensure survival under selective pressure.

The mechanisms underlying plant TGN remodeling and diversification are yet largely unknown. A subset of TGNs populated by TGNap1 tracks along MT. TGNap1 binds MT and is required for the maintenance of the budding, abundance and movement of a TGN subpopulation. Therefore, TGNap1 likely functions as a linker between a subset of TGN membranes and the MT. Such association is required for the budding of GI-TGNs from GA-TGNs and for the movement of TGNs that may precede fusion events of TGN-derived secretory vesicles with the PM or GA-TGNs. This hypothesis is predicated upon the evidence that, in addition to hampering trafficking routes, the loss of TGNap1 and chemical depletion of MT cause TGNs clustering and increase abundance of TGN-positive structures. In this model, the loss of a MT-linker such as TGNap1 or of MT may disrupt GI-TGN budding, leading to the observed increase in the size of the TGNs. The verified increase in the TGN vesicle number may be due to a reduced fusion of TGNap-1 vesicles with target membranes leading to disruption of traffic flow. A mammalian sequence homolog of TGNap1, KIAA1468, is a putative tether at the recycling endosome (RE) and TGN, promoting non-vesicular cholesterol transport between these two organelles^22^. In plants, the identity of the RE is yet undefined making not possible to ascribe a function to TGNap1 at a RE equivalent. It is also possible that TGNap1 has acquired plant-specific functions, as supported by the evidence that KIAA1468 functions in the transport of a small molecule while TGNap1 is involved in the trafficking and biogenesis of an organelle.

Plant organelle dynamics and post-Golgi traffic are believed to occur mainly on actin^15^. We demonstrated that the movement of a subpopulation of TGNs depends on MT. Albeit to a minor extent compared to oryzalin, latrunculin B treatment causes clustering of TGNap1-associated TGNs and a reduction of TGN movement, supporting that actin also partially contributes to the movement of the plant TGNs, consistently with the evidence that the TGNs positive for the putative actin-binding protein HLB1 track on actin^5^. Therefore, actin and MT-guided processes may converge on a subpopulation of TGNs where TGNap1 is localized with other proteins including HLB1; however, MT-guided processes may operate on a subpopulation of TGNs independently from actin.

TGNap1 has no motor function domains, supporting the hypothesis that TGNap1 may dynamically bridge TGN membranes with MT through the formation of a transient tether with the TGN. The tether may facilitate a close apposition of the TGN membrane to the cytoskeleton and the role of motor proteins in organelle translocation. Analogously, SYP73, a plant-specific ER-localized actin-binding protein, likely bridges dynamically the ER with actin thus facilitating the propelling action of myosin-XI^56^. Independently from the mechanism of action of TGNap1, our findings that post-Golgi traffic depends partially on MT and MT-binding proteins of the TGN constitute the foundations for an innovative model for endomembrane dynamics and function in plant cells that takes into account a significant contribution of MT.

Virtually nothing is known about how the specificity of the targeting of proteins and their permanency at TGN is regulated in plant cells. Similarly, knowledge on the identity and function of proteins complexes at the plant TGN is scarce^57^. Yeast and mammalian YIP homologs interact with Rab-GTPases and function in trafficking through yet largely unknown mechanisms^58–61^. In plants, similarly for YIP4, the function of Rab6 is yet unknown. YIP4 proteins, which are membrane anchored, interact with Rab6 and TGNap1, which also interact with each other and exchange on and off the TGN membranes from the cytosol. The cellular abundance of Rab6 has a significant bearing on the dynamics of TGNap1 on and off the TGN, and the loss of YIP4 alters the state-state association levels of Rab6 and TGNap1 with the TGN. Therefore, the verified YIP4-protein interactions regulate the temporal association of TGNap1 and Rab6 with the TGN, a role that is also contributed by Rab6 on the dynamics of TGNap1. These interactions may underlie a functional ternary complex or temporally-distinct binary protein–protein interactions. Unlike Rab6, TGNap1 interacts with MT, and the *TGNap1*, *YIP4*, and *Rab6* loss-of-function mutants are hypersensitive to oryzalin, supporting the occurrence of a functional interaction of Rab6 and YIP4 with the MT cytoskeleton that does not occur through a direct interaction with Rab6 but likely through TGNap1.

Metazoan Rab6 is required for the fission of Rab6-positive transport carriers from Golgi/TGN membranes^62^ and, in the GTP form, it interacts with golgin GCC185^63^, which is localized to a region of the TGN and plays a critical role in maintaining the organization of the Golgi apparatus^64^. These findings and the evidence that metazoan Rab6 interacts with Rab6-interacting protein 1 (R6IP1) with no nucleotide specificity^65^ support a diversification for the interaction requirements between Rabs and their partners, which may be dictated by the protein conformation specified by the nucleotide binding. We found specificity of the Rab6-TGNap1 interaction with the inactive Rab6. Based on the widely accepted model that active Rabs interact with their effectors, TGNap1 is unlikely a Rab6 effector. The different cycling rates of TGNap1 and Rab6 on and off the TGN membranes support this hypothesis as similar dynamics on and off membranes would be expected for a GTPase and its effector^66^. Therefore, the verified binding of TGNap1 to Rab6 is transient, and TGNap1 likely executes functions at the TGNs independently from a prolonged association to Rab6. Upon activation at the TGN Rab6 may be released from TGNap1 and interact with other proteins. Although additional Rabs may participate in the function of TGNap1, or a complete Rab6 knockout may have a stronger phenotype, the interaction of Rab6 and TGNap1 with YIP4 and the shorter permanency of TGNap1 on the TGN in a *rab6* mutant support that the interaction of inactive Rab6 may stabilize the docking of TGNap1 on the TGN. This is reinforced by a reduced mobile fraction of TGNap1-YFP in *yip4ab*, due to the lack of YIP4 proteins that influence Rab6 recycling at the TGN. For instance, the increased TGNap1 cycling on and off membranes may result from non-productive interactions of TGNap1 with the TGN membrane ensuing from an increase of Rab6-GDP-TGNap1 complexes on the TGN membrane in *yip4ab*, and consequent saturation of TGNap1-binding sites on the TGN. The evidence that Rab6 dynamics are unaffected by TGNap1 availability discards the alternative model that TGNap1 is required for Rab6 distribution and cycling at the TGN. Through an interaction with MT, TGNap1 may then control TGN budding and trafficking (Fig. 8d). The evidence that TGNap1 partially retains the ability to bind the TGN membrane in the *rab6* and *yip4* mutants argues that, analogously to golgin GCC185^63^, which binds to multiple GTPases^67^, TGNap1 binds the TGN membrane also through yet-unidentified proteins. Nonetheless, the functional correlation of TGNap1 with MT explains how a depletion of Rab6 or YIP4 causes hypersensitivity to induced MT-depolymerization. Therefore, YIP4, Rab6, and TGNap1 form a MT-dependent machinery required for the biogenesis and function of a subset of TGNs where TGNap1 plays a bridging role for MT and the TGN.

## Methods

### Plant material, growth conditions, and isolation of *tgnap1–1*

Plants of the *Arabidopsis thaliana* Col-0 and Landsberg *erecta* L*er* ecotypes and *Nicotiana tabacum* used in this study were cultivated at 21 °C under a 16 h light/8 h dark regime, and 23 °C 18 h light/18 °C 6 h dark regime, respectively. Analyses were performed in homozygous Col-0 plants either WT background or in stable transformants expressing the indicated constructs. The lines were obtained either through transformation by floral dip followed by antibiotic selection^68^ or by crosses with parental marker lines. *TGNap1* was isolated through a forward genetic screen on ethylmethansulfonate (EMS) mutagenized homozygous *A. thaliana* Col-0 expressing SEC-RFP^24^, a chimeric bulk-flow reporter based on the fusion of a monomeric red fluorescent protein 1 (mRFP)^20^ to a cleavable sporamin signal peptide. Mapping by next-generation sequencing^69^ was done on a mapping population created by crossing the *A. thaliana* Col-0/SEC-RFP mutant (M3 generation) with wild-type L*er*. Mapping was performed on 100 F2 individuals showing the SEC-RFP retention phenotype and the same number with wild-type phenotype. Once the regions with higher allele frequency difference on the chromosomes were identified, the putative candidates were narrowed down using the classical fine mapping, and then selecting the genes with the EMS canonical mutations (C to T changes). The *TGNap1* gene was selected upon mutant complementation. *Arabidopsis* seedlings were grown in 0.5x MS + phytagel media at 21 °C under a 16 h light/8 h dark regime. The PIN2:PIN2GFP and DR5-GFP lines were obtained from Dr. J. Friml. The *yip4ab* mutant was obtained from Dr. R.P. Bhalearo; KNOLLE-GFP, RabA2a-YFP were obtained from Dr. G. Drakakaki and HLB1-mCherry line was obtained from Dr. E. Blancaflor.

### Plasmid for protein expression in vivo and in vitro

Wild-type and mutated *TGNap1* were amplified and subcloned by standard approaches. Untagged WT and mutant *TGNap1* genes were amplified from cDNA of WT Col-0 and *tgnap1–1*, respectively, and subcloned for CaMV35S-driven expression between the ASCI and XBAI restriction sites in the multiple cloning site (MCS) of a pFGC5941 binary vector, which was modified to remove the CHSA intron present in the MCS. The cDNAs were also subcloned into the binary vector pEARLY-Gate 101 to generate YFP fusions at the C-terminus of cDNAs. The pET28b vector was employed to express recombinant TGNap1 and TGNap1T as His6 fusions at the N-terminus of the proteins, using the restriction sites *Nco*I and *Xho*I. For Rab6 cDNAs in the WT (empty), GDP and GTP were produced; GDP and GTP mutations were introduced as in the manuscript from Johansen et al.^42^ and were cloned as GST-fusions at the N-terminus in pGEX5 X-1 plasmid for in vitro interaction analyses; the restriction enzymes sites used were *BamH*I and *Xho*I. Primers were obtained by custom oligonucleotide synthesis (Invitrogen). All constructs were confirmed by sequencing. All primer sequences for those constructs are listed in Supplementary Table 1.

### Homology searches

The phylogenetic tree was generated through Homologene searches (https://www.ncbi.nlm.nih.gov/homologene), which identifies the species with the *TGNap1* homolog in the genome.

### RNA extraction and PCR amplification and real-time analysis

RNA was extracted using the NucleoSpin RNA plant Kit (Macherey-Nagel; http://www.mn-net.com) and either Taq or PFU polymerase was used for amplification of transcripts. Following mRNA extraction, reverse transcription PCR (RT-PCR) amplification of transcripts was carried out using 0.2 mM dNTPs, 0.2 μM primers, and 1 unit of Taq polymerase (Promega, http://www.promega.com), to establish transcript abundance; *Ubiquitin10* (*UBQ10*) was used as reference gene. For cloning, PCR amplification was performed with high-fidelity PFU following the manufacturer’s (Biolabs) instructions.

For quantitative real-time analysis, extracted RNA was used to synthesize cDNA using the iScript^TM^ cDNA Synthesis Kit following the manufacturer’s (BIO-RAD) instructions. Real-time PCR was performed using Fast SYBR Green master mix (Applied Biosystems) and a 7500 Fast-Real-time PCR system machine (Applied Biosystems). Primers were selected using Primer-Blast (http://www.ncbi.nih.gov/tools/primer-blast/) and validated though analysis of their melting curve. Experiments were performed in triplicate of three independent biological replicates. Expression level was determined by ΔΔ2*c*_t_ method^70^. The levels of gene expressions were normalized to *UBQ10*. Graph and statistical analysis was performed using Graph-Pad Prism (Graph-Pad Software) using paired ratio *t*-test.

### Confocal laser scanning microscopy

Acquisition of the confocal microscopy images was performed in non-saturating levels of pixel fluorescence intensity with an inverted laser scanner confocal microscope Nikon A1RSi. The fluorescent proteins used in this study were blue-shifted GFP (GFP5), EYFP (Clontech, http://www.clontech.com/), CFP, mCherry and monomeric RFP (mRFP)^20^. Imaging of these fluorescent markers^24^ presented in this work are representative of at least five independent experiments. The quantification analyses of pixel fluorescence intensity were performed on raw data. Post-acquisition handling of all images was realized exclusively for presentation, using the linear LUT function of the microscope software. The LUT function provides a non-destructive contrast enhancement of the image that improves the contrast and brightness by modifying the dynamic intensity of regions with poor contrast (https://www.gvsu.edu/cms4/asset/8FCAC028–902A-3EFC-5137403A360C8843/user_guide_nis-elements_ar.pdf). The changes applied with this function do not interfere with or alter the original data^71^. Within each experimental group, a linear auto-scale function with identical LUT settings was applied to enable a comparison within the dataset.

For FRAP experiments^72^, the average number of bleaching events used for the analysis was 52, specific number of independent data points is listed in the figures and/or figure legends. The FRAP protocol was performed on TGN structures in root epidermal cells of 6 DAG plants grown in vertical position on 0.5x MS + 1.2% agar. Student’s two tailed *t*-test was used for statistical analysis, assuming equal variance and data with *p*-value < 0.05 were considered significant.

For the FRET experiments, the donor was excited at 443 nm and light was collected at 468–503 nm; the acceptor was excited at 514 nm and the emitted fluorescence was collected between 570 and 620 nm. For the FRET pairs we used YFP for the YFP-Syp61 fusion; YFP-YIP4A and YFP-YIP4B and TGNap1-CFP. After sequential collection of CFP and YFP signals (pre-bleach), YFP (acceptor) was bleached for 10 s, and CFP signals were collected (post-bleaching acquisition). The energy transfer efficiency (*E*_FRET_) between the paired proteins was calculated based on the alteration in fluorescence intensity of the donor before (pre-bleach) and after photobleaching (post-bleach). The percentage change of the CFP intensity directly before and after bleaching was analyzed as *E*_FRET_ = (CFP_after_ – CFP_before_)/CFP_after_*100^73^. For analyses of fluorescence intensity profiles, the NIS element R intensity profile application of Nikon software was adopted. For PIN2:PIN2-GFP fluorescence analyses the number of roots analyzed for F.I. measurements was 27 for each genotype. Vacuole F.I were performed using a selected ROI for WT cell *n* = 459 and *tgnap1–2* cell *n* = 459. To measure Max intensity projections for DR5-GFP fluorescence analyses were carried out on 11 WT roots and 10 *tgnap1–2* roots. The data were produced either in cotyledon epidermal cells and primary root apex of 10-days-old stable Arabidopsis Col-0 transformants or in *N. tabacum* leaf epidermal cells. The latter were imaged exclusively for Supplementary Figs. 10A and 11A.

### Tobacco transient transformation

Protein transient expression was performed using 4-week-old *N. tabacum* (cv Petit Havana) plants and *Agrobacterium tumefaciens* (strain GV3101; OD_600_ = 0.05), according to an established protocol^74^.

### Lateral root density and auxin inhibitor response

Plants where grown vertically on 0.5x MS Agar for 5 days from germination and then transferred on 0.5x MS Agar containing 40 nM IAA and 1 μM IAA. Plants were grown for additional 5 days protected from light to minimize the IAA photodamage. Lateral root density was calculated measuring the total root length and dividing this length by the number of lateral roots formed. For auxin inhibitor responses, plants where grown vertically on 0.5x MS Agar for 3 days, then transferred on 0.5x MS Agar media containing 10 μM 1-*N*-Naphthylphthalamic acid (NPA), root were imaged 5 days after treatment and effect on elongation was measured as percent elongation.

### Phylogenetic analyses and protein motif identification

ClustalW alignment and phylogenetic tree for TGNap1 were generated using Mac vector 11 Software. TGNap1 amino acid sequence motifs identification was performed using ELM (Eukaryotic Linear motif)^45^.

### Transmission electron microscopy

Immediately after harvesting, roots of ten DAG seedlings were fixed overnight in ice-cold 0.1 M cacodylate buffer, pH 7.2, containing 2.5% (v/v) glutaraldehyde. They were then washed in the same buffer, post-fixed in 1% OsO_4_ for 2 h at room temperature (RT) and washed again in cacodylate buffer, pH 7.2. After dehydration in a graded ethanol series samples were embedded in Spurr’s epoxy resin (Electron Microscopy Sciences, Hatfield, PA, USA). Ultrathin sections (70 nm thick) were cut on an RMC ultramicrotome (RMC, Tucson, AZ, USA) and mounted on 150 mesh formvar-coated copper grids (Electron Microscopy Sciences). Just before analysis, the sections were stained with uranyl acetate for 30 min at room temperature, washed with ultrapure water and stained 10 min with lead citrate and washed again with ultrapure water. Images were taken with a JEOL100 CXII instrument (JEOL USA Inc., Peabody, MA) equipped with Gatan SC1000 (Model 832) CCD camera (Gatan Inc., Pleasanton, CA, USA).

### Treatments with ConcA and cytoskeleton-destabilizing agents

Plants were grown vertically on 0.5x MS Agar for 3 days from germination and then transferred on plates containing the various chemical inhibitors. ConcA (Sigma Aldrich CS9705) 1 mM stock was prepared in DMSO and added to 0.5x MS Agar plates at a final concentration of 50 nM. Oryzalin (ChemService N12729) 100 mM stock was prepared in DMSO and added to 0.5x MS Agar plates at a final concentration of 0.15 and 0.3 μM^75^. Effect on root elongation for ConcA treatment and on root tip swelling for oryzalin treatment was evaluated after 5 days. Data for root length of *tgnap1–2* and WT after ConcA treatment (Fig. 3c, d) and root tip swelling after oryzalin treatment in Supplementary Fig. 10B were produced on the basis of ten seedlings/treatment per genotype. The same number of samples was used for treatment on *rab6* and *yip4ab* in Supplementary Fig. 10B. All the experiments were replicated at least three times with consistent results. Oryzalin treatment for secretory marker (SEC-RFP) and TGN marker (YFP-SYP61) localization in absence of microtubules was performed in liquid 0.5x MS, respectively, for 12 and 7 h using oryzalin concentration of 10 μM. Dependence of endosomal movement on cytoskeleton was tested with treatment with either 25 μM Latrunculin B (30 min) or with 10 μM oryzalin (1 h).

### FM4–64 time course, BFA, CHX treatment, BFA body counting

Six-day-old *Arabidopsis* seedlings were pulse labeled with FM4–64 by incubation in 0.5x MS medium with 2 μM FM4–64 (500 μM; stock DMSO) for 5 min, washed three times at room temperature, mounted, and observed after 5, 15, 60 mins. For BFA treatment, the seedlings were first incubated for 5 min with 2 μM FM4–64 then incubated 1 h in 50 μM BFA solution. Confocal microscopy analyses of the FM4–64-stained seedlings were performed on epidermal cells of the root tip. Cycloheximide was dissolved in DMSO at a concentration of 50 mM and used at a final concentration of 50 μM for 1 h in the dark and then treated incubated for 1 h in 50 μM BFA solution. BFA body counts in Fig. 2b were done in 105 cells for WT and 125 for *tgnap1–2*. For lines expressing PIN2::PIN2-GFP and incubated with BFA (same concentration as detailed above) the total number of cells analyzed in Supplementary Fig. 4E across ten roots was 61 for WT and 67 for *tgnap1–2*. The number of cell analyzed for PIN2::PIN2-GFP after treatment with CHX and BFA was 920 for the WT and 920 for *tgnap1–2*. In Supplementary Fig. 4F, the total number of cells analyzed was 901 for WT and 544 for *tgnap1–2*. The number of cells analyzed for BFA body measurement in Fig. 8c was 100 for the untreated (DMSO) WT seedling, and 80 for the seedlings treated with oryzalin.

### Yeast two-hybrid analyses

Yeast two-hybrid analyses were performed using the matchmaker LexA system following the manufacturer’s manual (ClontechYeast protocol No. PT3024–1). The coding sequence (CDS) of *TGNap1* was cloned into pGilda-GW bait vector and the CDSs of *Rab6*, *YIP4A* and *YIPB* were cloned into pB42AD-GW prey vectors using LR reaction (Invitrogen, USA) individually to test the interactions. For the YIPs, the N-cytosolic tail (aa 1–140) of YIP4A ((YIP4B(-TM)) or (aa 1–143) for YIP4B ((YIP4B(-TM)) were used. The CDS of RAB6 was cloned also in pGilda to test the interaction with YIP4A and YIP4B, primers are listed in Supplementary Table 1. Each bait-prey combination was co-transformed into yeast strain EGY48. Competent cell preparation and transformation were performed using the Frozen EZ Yeast Transformation II (Zymo Research T2001). Protein–protein interactions were tested by β-galactosidase assay. The plate pictures were taken after 3 days of incubation at 28 °C. For the assays, positive or negative controls were coexpression of pLexA-53 and pB42AD-T vectors or empty plasmid, respectively, (pGilda empty and pB42AD empty). For each bait and prey combination, the assays were repeated three times with consistent results.

### TGNap1 and TGNap1T purification and in vitro interactions

Recombinant protein production was carried out in *E.coli* BL21. His-TGNap1 and His-TGNap1T were expressed in *E.coli* BL21, incubated with recombinant GST-Rab6 fusions and fixed on a resin for GST binding (PrepEase Protein purification Glutathione Agarose 4B USB). RAB6GTP was supplemented with GTPγS (Sigma). Immunoblot analyses and quantification^76^ of TGNap1 abundance on membrane were performed using anti-His serum (Santa Cruz Biotechnology Cat# SC-804) using a diluition of 1:2000 with an incubation time of two hrs. GST-Rab6 was detected using anti-GST serum (thermofisher scientific cat# A-5800) using a dilution 1:3000 with an incubation of 2 h. The TGNap1-Rab6 assay was repeated three times with consistent results. For MT interaction analyses, recombinant His-TGNap1T was expressed as before and purified (His-tagged protein purification Resin PreapEase USB). The assay was performed as recommended in the MT-Binding Protein Spin-Down Assay Biochem Kit Manual (Cytoskeleton Cat#BK029). Protein samples were loaded on a 12% SDS page gel and stained with imperial protein stain (Thermo Scientific # 24615).

### TGNap1-YFP protein extraction and western blot analyses

Two-week-old seedlings of Col-0 stable transformants expressing 35s:TGNap1-YFP were homogenated at 4 °C in PBS 1x buffer supplied with 1 mM phenylmethylsulfonyl fluoride, and 1x P9599 Protease inhibitors. The homogenate was then centrifuged at 5000 × *g* for 5 min at 4 °C. GFP antibody (abcam Ab290) was used at 1:3000 dilution and incubated for 3 h (see Supplementary Fig. 2G).

### Pull-down assay and LC/MS/MS analysis

Protein pull-down was performed using the Dynabeads Protein A Immunoprecipitation kit (ThermoFisher Scientific Cat#: Catalog no. 10006D). Protein extracts were isolated from 2-week-old *Arabidopsis* seedlings overexpressing TGNap1-YFP and from untransformed Col-0, which were homogenized in lysis buffer (10 mM Tris-HCl, 150 mM NaCl, 0.5 mM EDTA, 0.1% Triton, 1x protease inhibitor). The homogenates were centrifuged at 4 °C at 10,000 × *g* for 10 min, the supernatant isolated and incubated with the dynabeads protein A conjugated to the anti-GFP serum. After incubation, the beads were separated from the supernatant and washed using washing buffer. Proteins were eluted applying elution buffer to the beads. Eluted proteins were digested in solution, as follows: ammonium bicarbonate was added to each sample to 50 mM. Dithiothreitol was then added to 5 mM and the solutions incubated at 56 °C for 30 min. Iodoacetamide was added to 7 mM, and samples were incubated for 30 min at room temperature. Trypsin (200 ng) in 50 mM ammonium bicarbonate was then added and the mixtures were incubated for 15 h at 37 °C. The final volume of each solution was 300 µL. The solutions were acidified with 5% formic acid and centrifuged at 14,000 × *g* to remove any particulate. Peptide supernatants were removed and concentrated by solid phase extraction using c18 StageTips^77^. Purified peptide eluates were dried by vacuum centrifugation and re-suspended in 2% acetonitrile/0.1%TFA to 25 µL. Then 5 µL of this solution were automatically injected by a Thermo (www.thermo.com) EASYnLC 1000 into a Thermo Acclaim PepMap RSLC 0.075 mm × 150 mm C18 column and eluted over 60 min with a gradient of 2% Buffer B to 30% Buffer B in 48 min, ramping to 100% Buffer B at 50 min and held at 100% Buffer B for the duration of the run (Buffer B = 99.9% Acetonitrile/0.1% Formic Acid) at a constant flow of 300nL/min. Eluted peptides were sprayed into a ThermoFisher Q-Exactive mass spectrometer (www.thermo.com) using a FlexSpray spray ion source. Survey scans were taken in the Orbitrap (35000 resolution, determined at *m/z* 200) and the top ten ions in each survey scan are then subjected to automatic higher energy collision induced dissociation (HCD) with fragment spectra acquired at 17,500 resolution. The resulting MS/MS spectra were converted to peak lists using Mascot Distiller, v2.5.1 (www.matrixscience.com) and searched against the TAIR, v10, *A. thaliana* protein database (downloaded from www.arabidopsis.org) and appended with common laboratory contaminants (downloaded from www.thegpm.org, cRAP project) using the Mascot searching algorithm, v 2.5. The Mascot output was then analyzed using Scaffold, v4.7.3 (www.proteomesoftware.com) to probabilistically validate the protein identifications. Among the candidates, we selected proteins for which the number of peptides detected was ≥ 2.

### Ruthenium Red staining

WT and *tgnap1–2* seeds were soaked in 50 mM EDTA solution for 1 h at room temperature with shaking followed by 1-h incubation in 0.01% (w/v) Ruthenium red solution. Seeds were imaged using a Zeiss Axio Imager Upright Microscope and measure using the AxioVision LE64 software. Measurements were performed on 215 seeds for the WT and 215 seeds for *tgnap1–2*.

### Hemicellulose analyses

A 60 mg aliquot of dry biomass from 2-week-old etiolated hypocotyls was ball milled with the iWall grinding and feeding robot at the MSU Great Lakes Bioenergy Research Center Cell Wall Facility. The milled material was used to prepare the alcohol insoluble residue (AIR) through aqueous and solvent washes to extract the soluble components (soluble sugars, proteins, lipids, pigments, DNA, and RNA). The AIR was then incubated with amylase and pullulanase and subsequently washed with water to remove starch, yielding lignocellulosic material for cell wall analysis^78^. The isolated lignocellulosic cell wall material was dried and weighed into three, 2 mg technical replicates to assay the matrix polysaccharide composition. The polysaccharide composition was analyzed via GC-MS (Agilent 7890A GC / 58975C MS) after a 2 M trifluoroacetic acid (TFA) hydrolysis and subsequent alditol acetate derivatization of the neutral monosaccharides present the hydrolysate.

## Supplementary Information

Supplementary Information

Supplementary Movie 1

Supplementary Movie 2

Description of Additional Supplementary Files

Source Data

Reporting Summary

## Data Availability

The authors declare that the data supporting the findings of this study are available within the Article and its Supplementary files, as detailed in the Reporting Summary. All relevant data are available from the authors upon request with no restrictions. The mass spectrometry proteomics data relative to TGNap1 co-IP and its control from Col0 have been deposited to the ProteomeXchange Consortium via the PRIDE partner repository respectively with the dataset identifier PXD011581 and dataset identifier PXD011578. Data-points for the experiments are included in the Source Data file.
