# Supplementary material for: TGNap1 is required for microtubule-dependent homeostasis of a subpopulation of the plant trans-Golgi network
Source: Nat Commun. 2018 Dec 14;9:5313. doi: 10.1038/s41467-018-07662-4 (PMC6294250; doi:10.1038/s41467-018-07662-4)
Supplement: Supplementary file 1 — Supplementary Information [file 41467_2018_7662_MOESM1_ESM.pdf]

Supplementary Figures

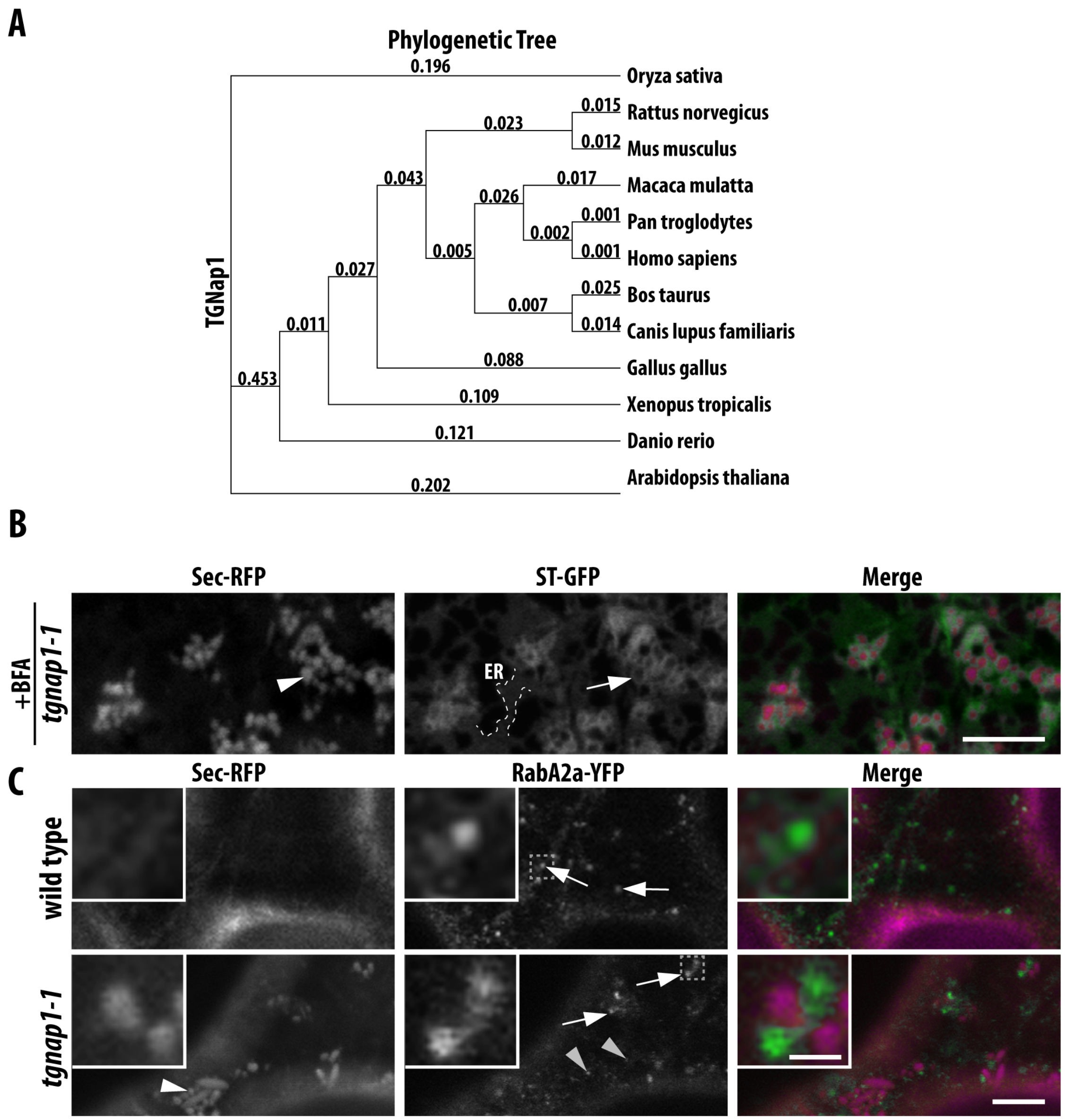

Supplementary Figure. 1 related to Figure1. TGNap1 is conserved in multicellular organisms, and its mutation affects secretion and TGN morphology

A. Phylogenetic analysis of TGNap1 across the indicated genomes represented in a Neighbor Joining Tree constructed with uncorrected P distances. No sequences with significant homology were identified in yeast. **B** Live-cell confocal images of cotyledon epidermal cell of *tgnap1-1* expressing the Golgi marker ST-

GFP treated with BFA. An arrowhead indicates a Sec-RFP globular structure surrounded by the ST-GFP marker (arrow) relocated to the ER (dotted line highlighting part of ER cisternae). **C** Live-cell confocal images of cotyledon epidermal cell showing aberrant morphology of the TGN/EEs highlighted by the marker RabA2a in *tgnap1-1* compared to WT. Arrows show TGNs. The yellow dotted polygon refers to the TGNs visualized in the magnification inset. The white arrowhead in the Sec-RFP panel points to the globular structures typical of the *tgnap1-1* mutant and the orange arrowheads in the RabA2a-YFP panel point at smaller structures more abundant in the mutant compared to WT. Scale bars = 5 µm, inset scale bar = 1 µm.

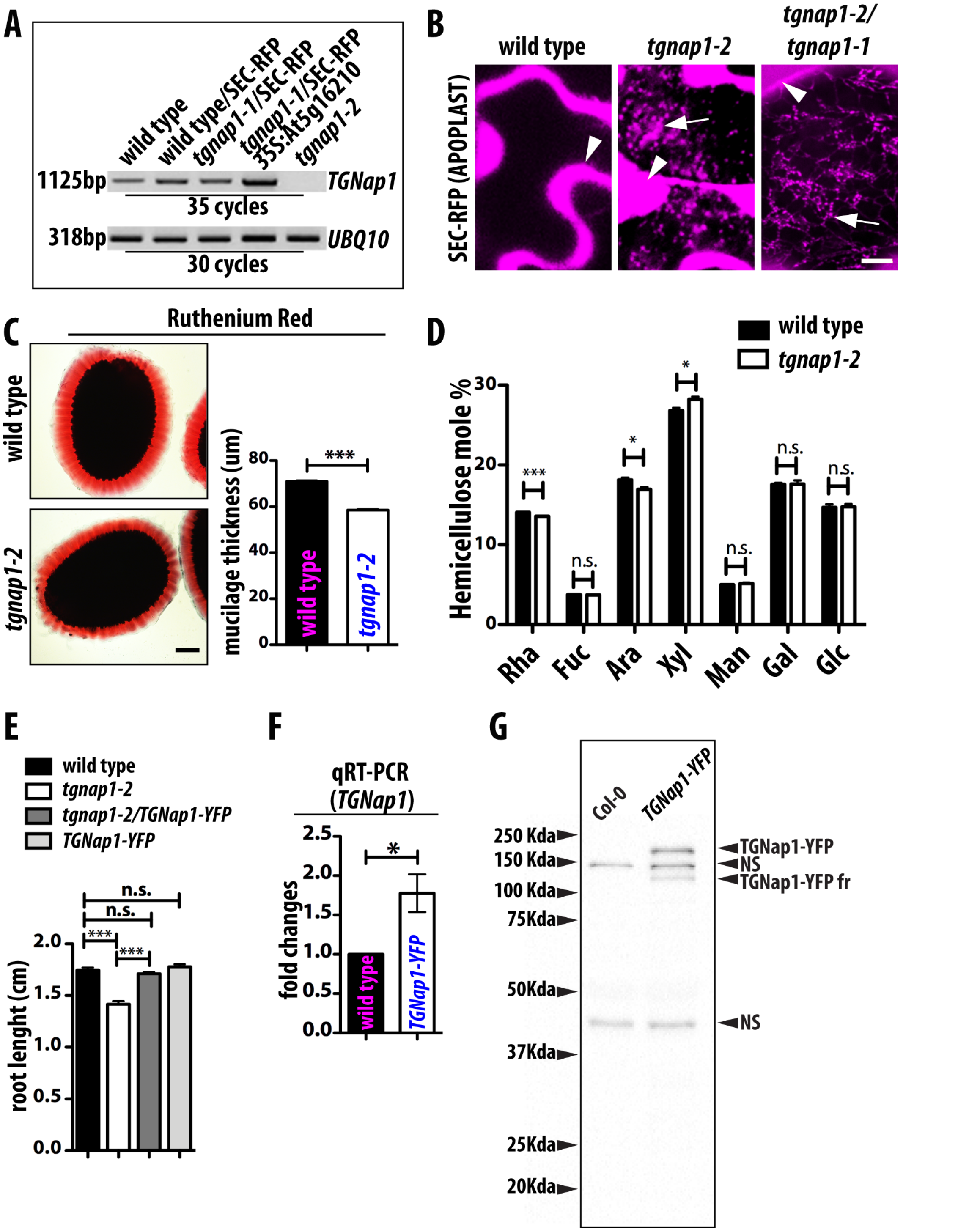

Supplementary Figure. 2 related to Figure1. The loss of TGNap1 compromises secretion of proteins and cell wall matrix carbohydrates

A. RT-PCR analysis of full-length *TGNap1* showing absence of transcript only in *tgnap1-2* **B.** Live-cell confocal images of cotyledon epidermal cells showing distribution of SEC-RFP in the extracellular environment (apoplast, arrowheads) in WT and *tgnap1-2*, and *tgnap1-1* x *tgnap1-2* (F1 generation) with partial retention of SEC-RFP in intracellular globular structures in *tgnap1-2* (arrows). **C.** Ruthenium red mucilage staining and quantification on WT and *tgnap1-2* seeds. **D.** Hemicellulose composition of cell wall polysaccharides in hypocotyl. Student`s t test was applied. Error bars indicate SEM. *** P< 0.001; *0.01>P<0.05; n.s., not significant. **E.** Measurements of primary root length in WT, *tgnap1-2*, complemented *tgnap1-2* with TGNap1-YFP driven by the 35S CaMV promoter (*tgnap1-2*/TGNap1-YFP), and WT Col-0 plants expressing TGNap1-YFP driven by the 35S CaMV promoter (TGNap1-YFP). Measurements from three independent experiments. One-way Anova with Tukey post test was applied. Error bars indicate SEM. *** P< 0.001; **0.001> P< 0.01; n.s., not significant. **F.** qRT-PCR on WT and Col-0 overexpressing 35S::TGNap1-YFP. Student`s t test was applied. **G.** Full-length Western blot with anti-GFP on protein extracts from Col0 WT plants and a 35S::TGNap1-YFP line. Arrowheads on the left of panel indicate molecular; arrowheads on the right indicate top band relative to TGNap1-YFP (164 KDa), non-specific binding (NS) and a probable TGNap1-YFP fragmentation product (fr).

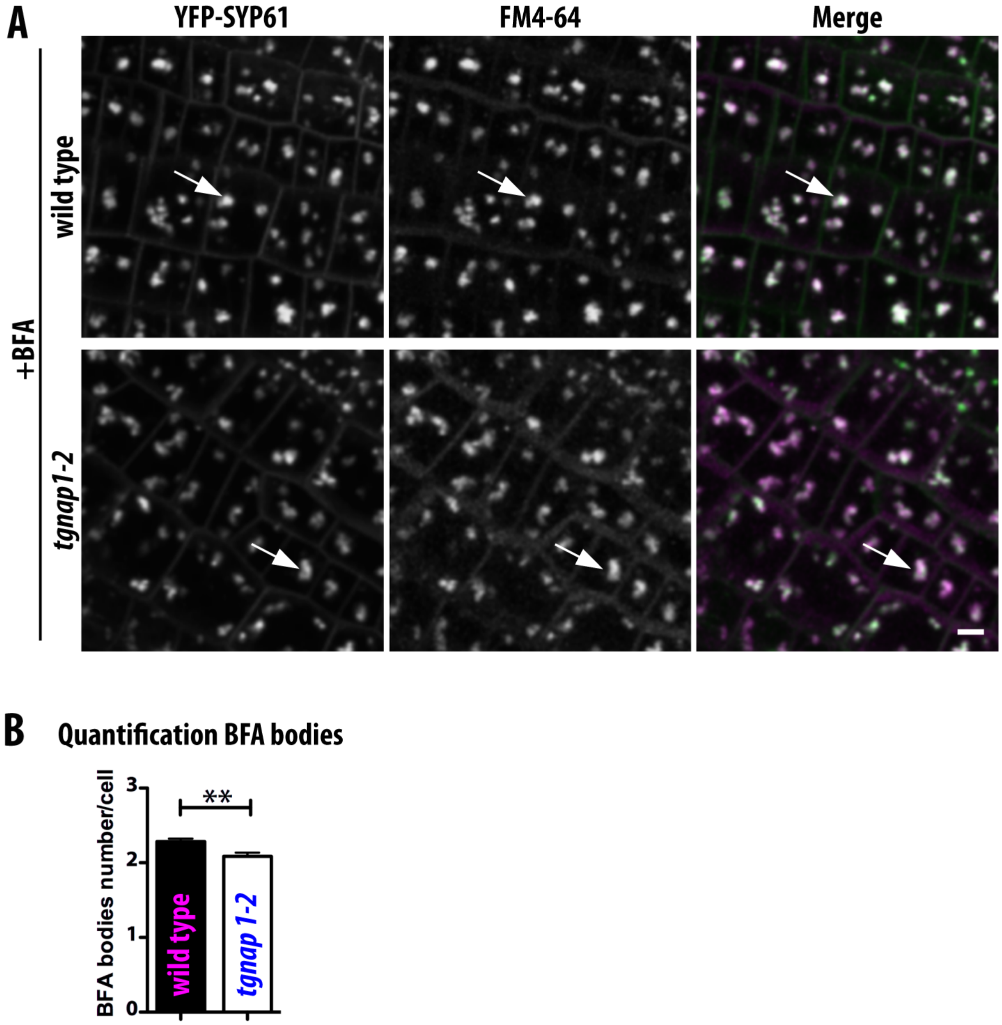

Supplementary Figure. 3 related to Figure 2. The loss of TGNap1 compromises endocytosis

A. Confocal images showing BFA bodies (arrowheads) in WT and *tgnap1-2* root cells expressing the TGN marker YFP-Syp61, pulse-labeled with FM4-64 for 5 min. **B.** Quantification of the number of BFA bodies/cell. WT cells (n=901), *tgnap1-2* cells (n=544). Student`s t test was applied. Error bars indicate SEM. *** P< 0.001; *0.01>P<0.05. Scale bar = 5 μm.

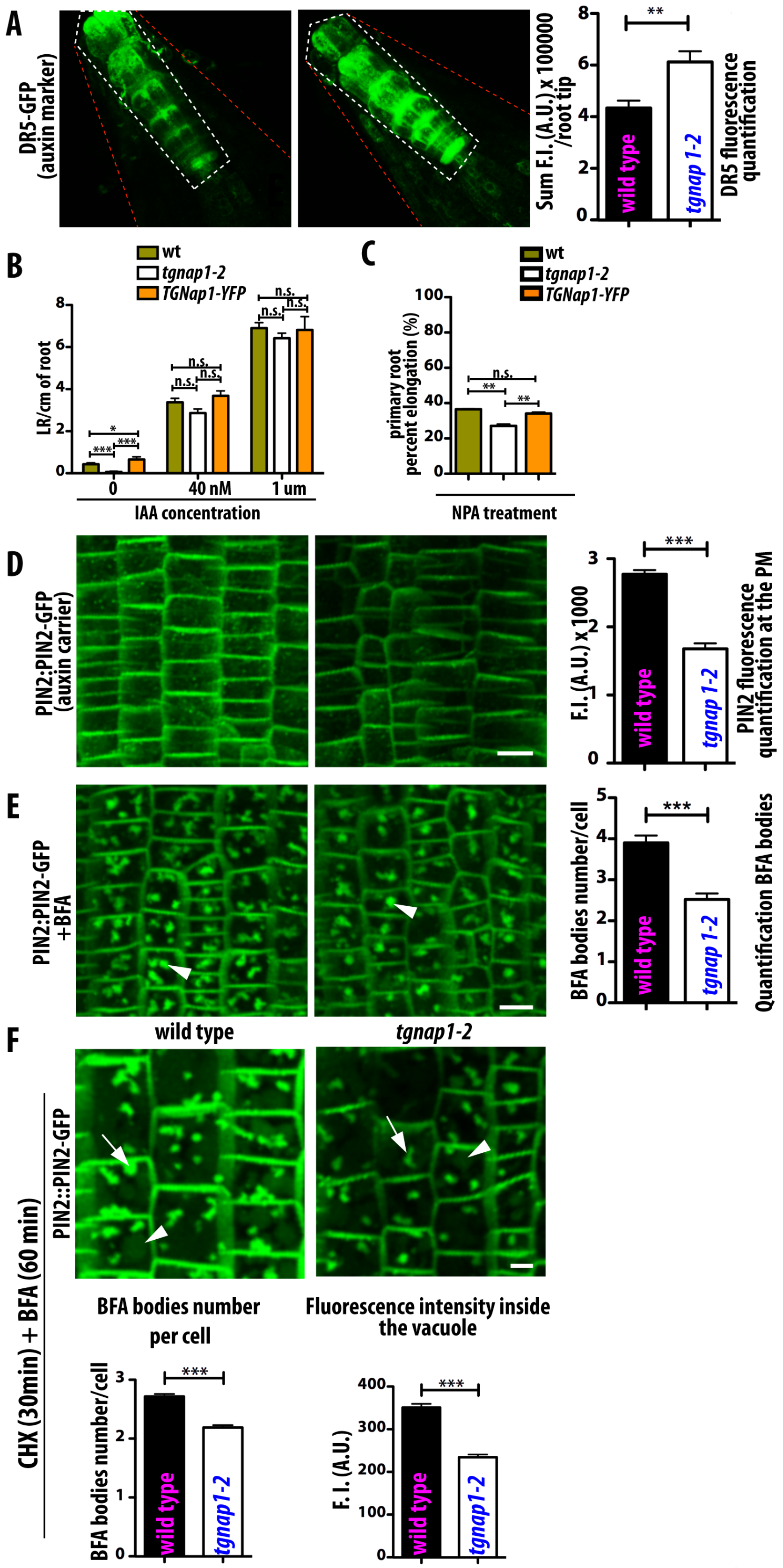
A
DR5-GFP
(auxin marker)
Sum F.I. (A.U.) x 100000 /root tip
**
wild type
tgnap1-2
DR5 fluorescence quantification
B
wt
tgnap1-2
TGNap1-YFP
LR/cm of root
IAA concentration
0
40 nM
1 um
n.s.
C
primary root percent elongation (%)
NPA treatment
D
PIN2:PIN2-GFP
(auxin carrier)
F.I. (A.U.) x 1000
***
PIN2 fluorescence quantification at the PM
E
PIN2:PIN2-GFP
+BFA
BFA bodies number/cell
Quantification BFA bodies
F
wild type
tgnap1-2
PIN2::PIN2-GFP
CHX (30min) + BFA (60 min)
BFA bodies number per cell
Fluorescence intensity inside the vacuole
F. I. (A.U.)

Supplementary Figure. 4 related to Figure 2. The loss of TGNap1 compromises endocytosis

A. Max intensity projection of serial confocal images (depth: 110 μm) of WT and *tgnap1-2* root tips (perimeter highlighted by red trace) showing DR5-GFP localization in the apical portion of the root (boxed by white trace). Measurements from three independent experiments with 10 seedlings each. **B.** Graph showing lateral root (LR) density quantification upon IAA treatment (40 nM and 1 μM) in WT, *tgnap1-2* and TGNap1-YFP overexpressing line. Measurements from three independent experiments. One-way Anova with Tukey post test was applied. **C.** Graph showing primary root elongation percentage upon 10 μM NPA treatment in WT, *tgnap1-2* and TGNap1-YFP overexpressing line. Measurements from three independent experiments. One-way Anova with Tukey post test was applied**. D.** Max intensity projection of optical slices (depth: 35 μm) of WT and *tgnap1-2* root cells expressing PIN2:PIN2-GFP. Quantification of GFP fluorescence intensity (F.I. expressed in arbitrary units, A.U.) at the PM is also indicated. Measurements from three independent experiments. Student`s t test was applied. **E.** Confocal images of WT and *tgnap1-2* root cells expressing PIN2:PIN2-GFP upon BFA treatment. Arrowheads: BFA bodies. Quantification of BFA bodies/cell is presented in the graph. WT cells (n=61), *tgnap1-2* cells (n=67). Student`s t test was applied **F.** Confocal images of WT and *tgnap1-2* root cells expressing PIN2:PIN2-GFP upon CHX and BFA treatment. Arrow: BFA bodies, Arrowheads: vacuoles. Quantification of BFA bodies/cell and fluorescence intensity inside the vacuole is presented in graph. Student`s t test was applied. Error bars indicate SEM; **$0.001 > p < 0.01$; *** $p < 0.001$. Scale bars = 5 μm.

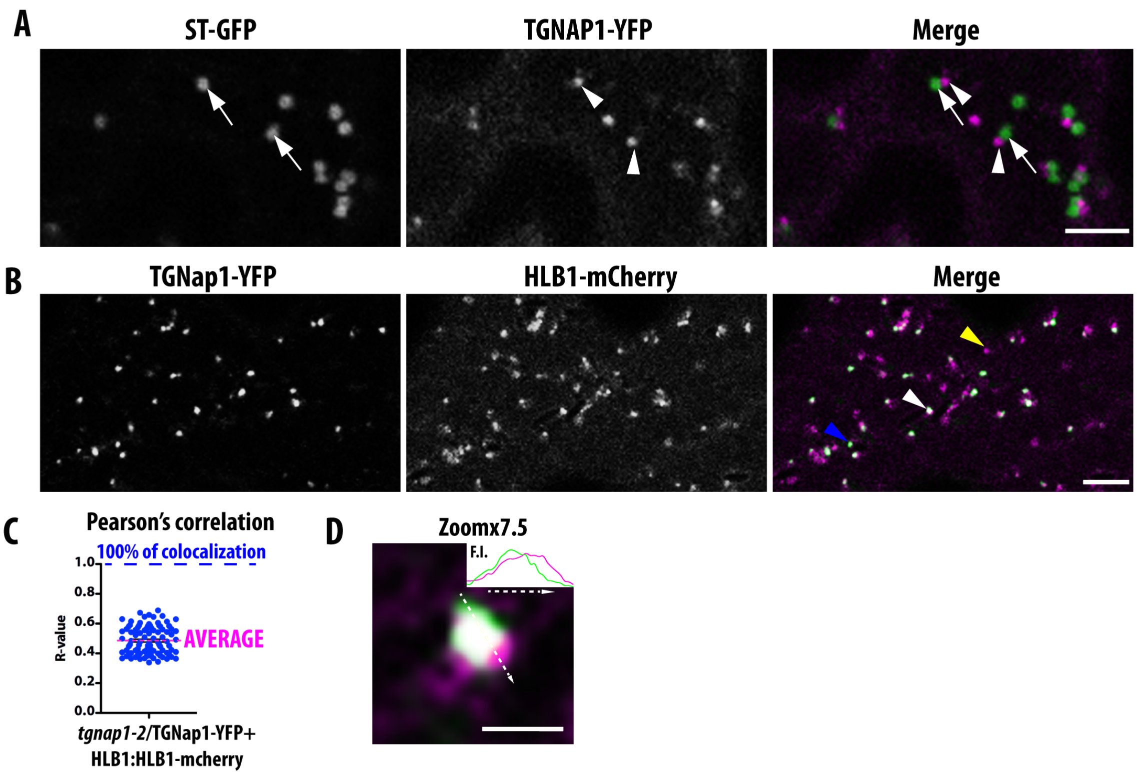

Supplementary Figure. 5 related to Figure 3. TGNap1 partially colocalizes with HLB1 at a TGN subpopulation but not at the Golgi

A. Live-cell confocal images of epidermal cells coexpressing TGNap1-YFP and the Golgi marker ST-GFP showing no colocalization. Arrows indicates Golgi labeled by ST-GFP; arrowheads indicate TGNs labeled by TGNap1-YFP. Scale bar = 5 µm. **B.** Live-cell confocal images of epidermal cells coexpressing TGNap1-YFP and the TGN marker HLB1-mCherry. A blue arrowhead indicates a TGN containing TGNap1-YFP; a white arrowhead indicates a TGN containing TGNap1-YFP and HLB1-mCherry; a yellow arrowhead points to TGN containing only HLB1-mCherry signal. Scale bar = 5 µm. **C.** Pearson's correlation coefficient (R-value) measurements for YFP/mCherry image pairs. **D.** Zoomx7.5 panel: 7.5x magnification of the dot marked by white arrow in the merge image **A.** Scale bar = 1 µm. The graphs in the insets represent pixel fluorescence intensity (F.I.) measurements for TGNap1-YFP (green) and HLB1-mCherry (red) in raw data,

across the length of the arrow delineated on individual TGN. The scans indicate non-complete overlap of the two signals.

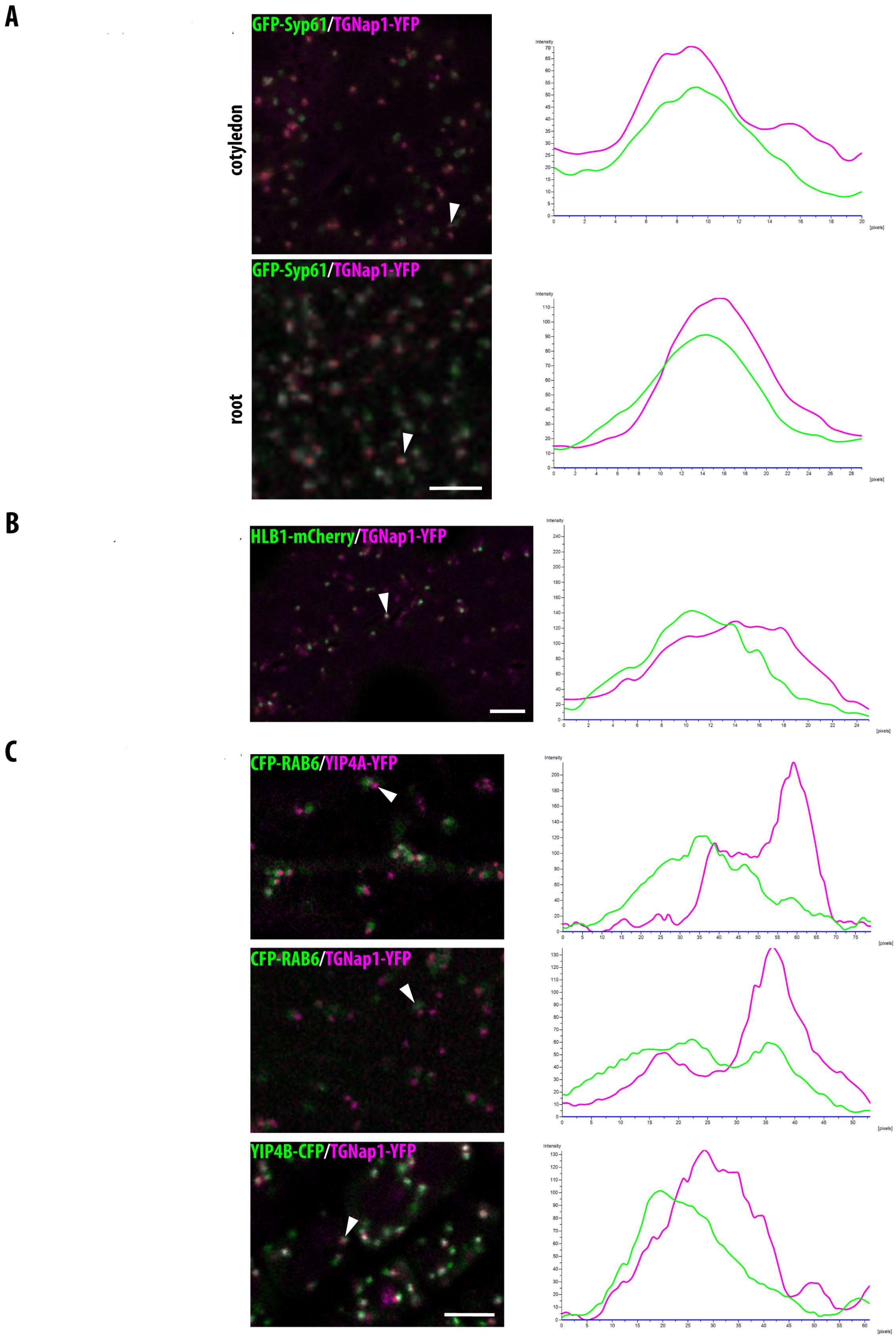
A
GFP-Syp61/TGNap1-YFP
cotyledon
GFP-Syp61/TGNap1-YFP
root
B
HLB1-mCherry/TGNap1-YFP
C
CFP-RAB6/YIP4A-YFP
CFP-RAB6/TGNap1-YFP
YIP4B-CFP/TGNap1-YFP
Intensity
[pixels]

Supplementary Figure. 6 related to Figure 3,4,5. Raw data imaging colocalization analyses and expression intensity profile

Live-cell confocal images of cotyledon epidermal cells or root epidermal cells expressing the markers used for quantification analysis. A. raw data related to Figure 3A; B. raw data related to Supplementary Figure 5B; C raw data related to Figure 4A,B,C. The corresponding graphs show the pixel intensity profiles of the two protein fusions. Intensity profile scale shows that the fluorescence levels (A.U.) are not saturated. Scale bars = 5 μm.

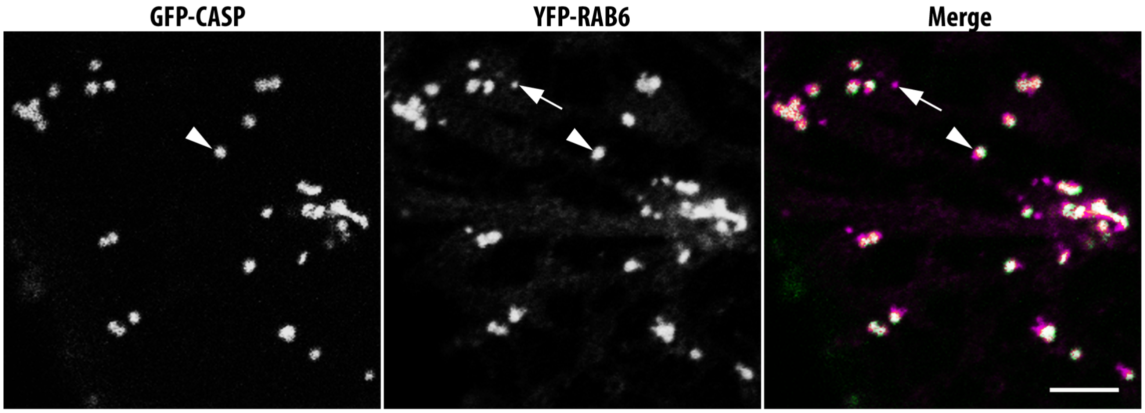

Supplementary Figure. 7 related to Figure 4. Rab6 localizes at Golgi

Confocal images of cotyledon epidermal cells expressing GFP-CASP and YFP-Rab6 showing that the two proteins colocalize (Golgi stacks; arrowheads). Rab6 additionally localizes to additional structures devoid of the Golgi marker (arrow). Scale bar = 5 μm.

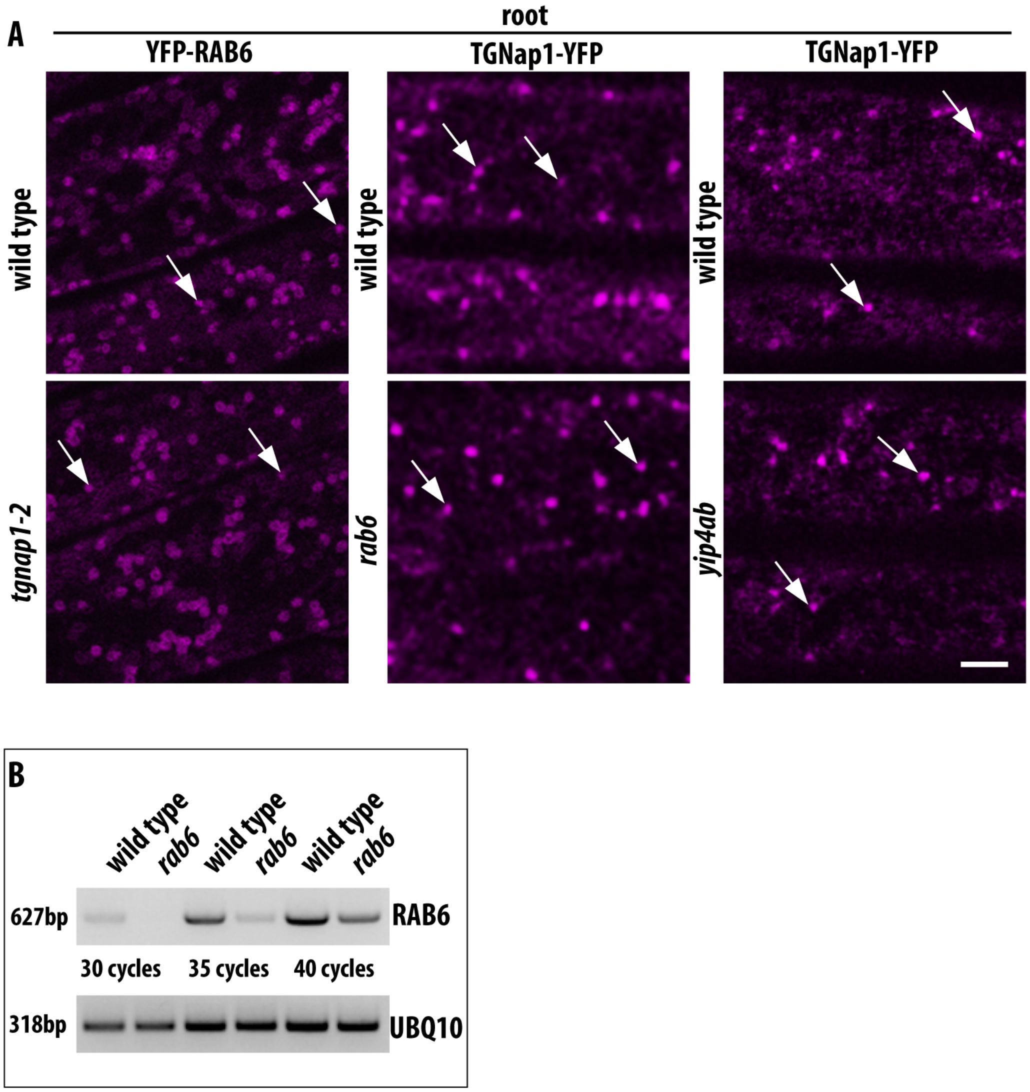

Supplementary Figure. 8 related to Figure 5. Subcellular distribution of YFP-Rab6 and TGNap1-YFP in WT and loss-of-function mutants

A. Live-cell confocal images of WT and *tgnap1-2* root epidermal cells expressing YFP-RAB6 and the subcellular distribution of TGNap1-YFP in either WT or *rab6* or *yip4ab*. Arrows indicate TGNs used for FRAP analyses. Scale bars = 5 µm. **B.** RT-PCR analysis (30, 35 and 40 PCR cycles) of full-length RAB6 showing reduced levels of transcript in *rab6* compared to WT.

Col-0	TGNap1-YFP		
0	122	TGNap1	AT5G16210
3	9	actin 7	AT5G09810
0	8	heat shock cognate protein 70-1	AT5G02500
0	9	heat shock protein 81-3	AT5G56010
0	7	calcium sensing receptor	AT5G23060
0	6	PATELLIN 1	AT1G72150
0	6	Transducin/WD40 repeat-like superfamily protein	AT2G16405
0	5	tubulin beta chain 4	AT5G44340
0	5	ATPase, V1 complex, subunit B protein	AT1G20260
0	5	ADP-ribosylation factor A1F	AT1G10630
0	4	PATELLIN 2	AT1G22530
0	4	tubulin alpha-3	AT5G19770
0	4	vacuolar ATP synthase subunit A	AT1G78900
0	3	vacuolar ATP synthase subunit E1	AT4G11150
0	2	vacuolar ATP synthase subunit C	AT1G12840
0	2	Clathrin heavy chain	AT3G08530

Color scale: 0 – 122, number of peptides

Supplementary Figure. 9 related to Figure 6. TGNap1 co-immunoprecipitation analysis showing the candidates identified with a number of peptides above two in TGNap1-YFP extracts only

List of proteins and heat map of the number of peptides detected by co-IP experiment for TGNap1-YFP (0 indicates absence of peptides).

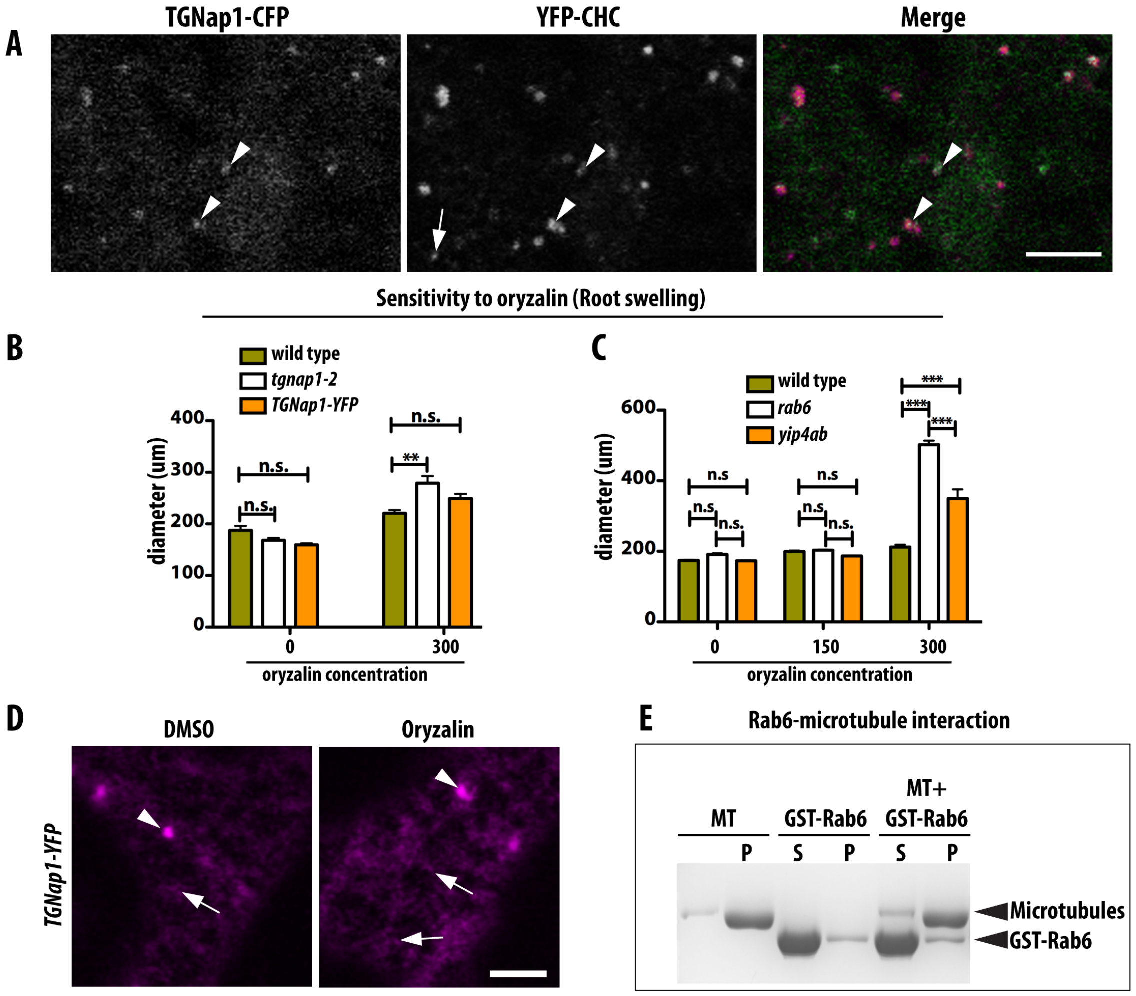

Supplementary Figure. 10 related to Figure 6. TGNap1 subcellular distribution, *tgnap1* sensitivity to oryzalin, and Rab6 inability to bind MT

A. Confocal images of tobacco epidermal cells expressing TGNap1-CFP and YFP-CHC showing that the two proteins colocalize (arrowheads). Arrow points to a TGNap1-CFP TGN that does not contain YFP-CHC. Scale bar = 5 μm. **B.** Measurements of the root swelling of oryzalin-treated seedlings indicate that, unlikely the TGNap1-YFP overexpressing line, *tgnap1-2* is more sensitive to oryzalin compared to the WT. Measurements from three independent experiments. **C.** Measurements of the root swelling of oryzalin-treated seedlings indicate that *rab6* and *yip4ab* are significantly more sensitive to the drug compared to WT. Measurements from three independent experiments. Error bars indicate SEM. *** P< 0.001; n.s., not significant. **D.** Confocal images of cotyledon

epidermal cells expressing TGNap1-YFP showing that the loss of MT alters the subcellular distribution of TGNap1-YFP. Scale bar = 5 µm. **E.** MT sedimentation assay with recombinant GST-RAB6 (MW= 50 Kda) and bovine brain MT (Microtubules MW= 55 Kda) showing that GST-RAB6 does not precipitate in the pellet (P) in the presence of MT in the reaction.

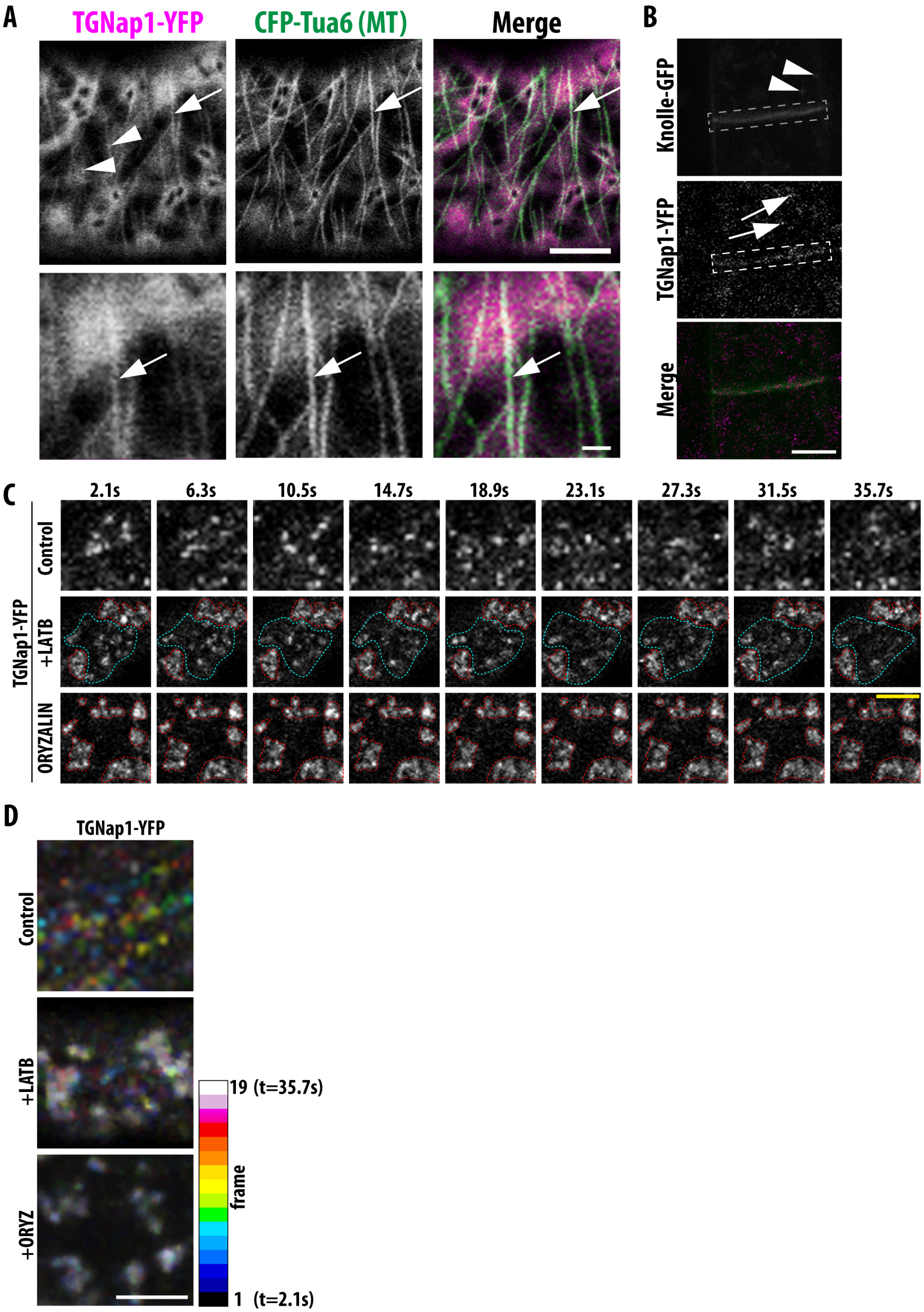

A
TGNap1-YFP
CFP-Tua6 (MT)
Merge
B
Knolle-GFP
TGNap1-YFP
Merge
C
2.1s
6.3s
10.5s
14.7s
18.9s
23.1s
27.3s
31.5s
35.7s
Control
+LATB
ORYZALIN
TGNap1-YFP
D
TGNap1-YFP
Control
+LATB
+ORYZ
19 (t=35.7s)
frame
1 (t=2.1s)

Supplementary Figure. 11 relative to Supplementary Figure. 6. Subcellular distribution of TGNap1-YFP in transient expression in the presence of CFP-Tua6, and of TGNap1-YFP-TGNs in the presence of cytoskeleton inhibitors

A. Confocal images of transiently transformed *Nicotiana tabacum* leaf epidermal cells expressing TGNap1-YFP in presence of the MT marker CFP-TUA6, showing a partial localization of TGNap1 to the cytosol, and MT (arrows). Arrowheads indicate localization of TGNap1 on punctae, likely TGNs. Top panel scale bar = 5 µm. Bottom panels are magnified areas pointed by arrows in main image; scale bar = 1 µm. **B.** Confocal images of root cortex cells during division expressing KNOLLE-GFP and TGNap1-YFP showing co-distribution to the cell plate (white dotted area), arrows and arrowheads point at structures that do not overlap. **C.** Images extracted from confocal microscopy time-lapse acquired on root cortex cells expressing TGNap1-YFP with no treatment, Lat B or oryzalin treatment showing largely immotile clusters (red-dotted areas). Scale bar = 5 µm. **D.** Compound panels are composite of 9 frames each, pseudocolored along a color gradient (see scale), captured at 2.1 secs intervals. White color (last frame) overlapping other colors denotes slow motility of the TGNs. Scale bar = 5 µm.

Supplementary Table1: Reagents and Primers

REAGENT or RESOURCE	SOURCE	IDENTIFIER
**Antibodies**		
anti-His	Santa Cruz biotechnology	sc-804
anti-GST	ThermoFisher scientific	A-5800
anti-GFP	Abcam	A290
**Bacterial and Virus Strains**		
Agrobacterium tumefaciens GV3101	n/a	n/a
E.coli BL21 DE3	n/a	n/a
**Chemicals, Peptides, and Recombinant Proteins**		
FM4-64	ThermoFisher scientific	T13320
BFA	Sigma	B5936
Oryzalin	Chem Service	N-12729
Latrunculin B	Calbiochem	428020
Cycloheximide	Bioshop	CYC003
Cacodylate buffer	Electron Microscopy Sciences, Hatfield, PA, USA	11654
Glutaraldehyde	Electron Microscopy Sciences, Hatfield, PA, USA	16220
OsO_4	Electron Microscopy Sciences, Hatfield, PA, USA	
Spurr's epoxy resin	Electron Microscopy Sciences, Hatfield, PA, USA	
Uranyl acetate	Sigma	
Lead citrate	Sigma	

**Critical Commercial Assays**		
Microtubule Binding Protein Spin down Assay Kit	Cytoskeleton	BK029
**Experimental Models: Organisms/Strains**		
Arabidopsis thaliana Col3 SAIL_31_C09	Tair	TGNap1 tDNA insertion line
Arabidopsis thaliana Col0 SALK_070151	Tair	Rab6 tDNA insertion line
Arabidopsis thaliana Col3		
Arabidopsis thaliana Col0		
**Oligonucleotides**		
atggacgcggagcgatcgtcgttgtgcaactttttcg	Invitrogen	At5g16210-5' for P101GW
gtgattttgtggctgtgtctcatcttgggtcttggc	Invitrogen	At5g16210-3' for P101GW
ggtgctaccatggacgcggagcgatcgtcgttgtgc	Invitrogen	At5g16210-5' for Pet28
gtggtgctcgaggtgattttgtggctgtgtctcatcttgg	Invitrogen	At5g16210-3' for Pet28
gtggcgcgccatggacgcggagcgatcgtcgttgtgc	Invitrogen	At5g16210-5`for PFGC
aggacgtctagattatttgtacaattcatccataccatgggtaatacc	Invitrogen	At5g16210-3`for PFGC
gtggtgctcgaggaggttcctcctctgatgctctgaggat	Invitrogen	At5g16210T-3`for pET28
ttagtgattttgtggctgtgtctcatcttgggtcttggc	Invitrogen	At5g16210-3`for pGILDA
atggctccggtctcggcactcgctaag	Invitrogen	At2g44610-5'for P104 GW or pVKH18-NcCFP GW
ttagtagccgcccatcggtgg	Invitrogen	At2g44610-3'for P104 GW or pVKH18-NcCFP GW
ggtgctggatccccatggctccggtctcggcactcgc	Invitrogen	At2g44610-5'for pGEX5x-1
gtggtgctcgagctaacaagagcatcctcctgattgctgc	Invitrogen	At2g44610-3'for pGEX5x-1
caatccgtcggcaagaattccatcattactcgatcc	Invitrogen	At2g44610-5'for T23N
gaatcgagtaatgatggaattcttgccgacggattg	Invitrogen	At2g44610-3'for T23N
ttgtgggatacagcaggcctagagcgattcaggagt	Invitrogen	At2g44610-5'for Q68L
actcctgaatcgctctaggcctgctgtatcccacaa	Invitrogen	At2g44610-3'for Q68L
atgtcacaaggcgatacagtaccgctt	Invitrogen	At2g18840-5' for pVKH18-NcCFP GW
tcaattgatggctatgatgagaaaacc	Invitrogen	At2g18840-3' for pVKH18-NcCFP GW
tcaatctctaagagccttcccaggatc	Invitrogen	At2g18840-3' for pB42-AD GW
atgtcgcacaacgatacgattccgctt	Invitrogen	At4g30260-5' for pVKH18-NcCFP GW

tcaattaatggcaatgattaagaagcc	Invitrogen	At4g30260-3' for pVKH18-NcCFP GW
tcaatccctaagtgctttcccaggatc	Invitrogen	At4g30260-3' for pB42-AD GW
tatgttgccaaacgaccctac	Invitrogen	LP primer for RAB6 tDNA insertion line
tttagggacatggtggaactg	Invitrogen	RP primer for RAB6 tDNA insertion line
ccacaaagtttgttggaggag	Invitrogen	LP primer for TGNap1 tDNA insertion line
tcagaaagaacatacaaagccg	Invitrogen	LP primer for TGNap1 tDNA insertion line
attttgccgatttcggaac	Invitrogen	BP primer for Salk t-DNA insertion line
gcttcctattatatcttcccaaattaccaataca	Invitrogen	BP primer for Sail t-DNA insertion line
tcaattctctctaccgtgatcaagatgca	Invitrogen	5' Ubi10
ggtgtcagaactctccacctcaagagta	Invitrogen	3' Ubi10
ttcctcacacgacagaacgg	Invitrogen	TGNap1 qRT_Fw
gtgcacgaatggcttcacag	Invitrogen	TGNap1 qRT_Rv
ggccttgtataatccctgatgaataag	Invitrogen	Ubi10 qRT_Fw
aaagagataacaggaacggaaacatagt	Invitrogen	Ubi10 qRT_Rv
**Recombinant DNA**		
pEarlyGateway101-TGNap1-YFP		
pVKh18EnGateway- CFP RAB6		
pEarlyGateway104-YFP-RAB6		
pVKh18EnGateway- CFP YIP4a		
pVKh18EnGateway- CFP YIP4b		
pET28-TGNap1T		
pGEX5x-1-RAB6		
pGEX5x-1-RAB6 GDP		
pGEX5x-1-RAB6 GTP		
pGILDA-TGNap1		
pB42AD- YIP4a		
pB42AD- YIP4b		
pB42AD-RAB6		
**Software and Algorithms**		
ImageJ	NIH	https://imagej.nih.gov/ij/
Graphpad Prism	Graphpad	https://graphpad.com

NIS-Elements AR	Nikon	https://www.nikoninstruments.com/Products/Software/NIS-Elements-Advanced-Research
AdobePhotoshop	Adobe	https://www.adobe.com/products/photoshop.html
