# Supplementary material for: TGNap1 is required for microtubule-dependent homeostasis of a subpopulation of the plant trans-Golgi network
Source: Nat Commun. 2018 Dec 14;9:5313. doi: 10.1038/s41467-018-07662-4 (PMC6294250; doi:10.1038/s41467-018-07662-4)
Supplement: Supplementary file 4 — Description of Additional Supplementary Files [file 41467_2018_7662_MOESM4_ESM.pdf]

Description of Additional Supplementary Files

File Name: Supplementary Movie 1.

Description: TGNap1-YFP tracks on MT labeled by CFP-Tua6.

File Name: Supplementary Movie 2.

Description: Biogenesis of GI-TGNs labeled by YFP-Syp61 in WT (leftSupplementary movie 2A) or tgnap1-2 background (right-Supplementary Movie 2B).
